# Cooperative breeding and the selection for information sharing among groupmates

**DOI:** 10.1007/s00265-025-03604-5

**Published:** 2025-06-17

**Authors:** Laure A. Olivier, Tim W. Fawcett, Andrew N. Radford, Andrew D. Higginson

**Affiliations:** 1 Centre for Research in Animal Behaviour, Washington Singer Laboratories, University of Exeter, Exeter, EX4 4QG UK; 2 School of Biological Sciences, Life Sciences Building, 24 Tyndall Avenue, Bristol, BS8 1 TQ UK

**Keywords:** Reproductive skew, Outside option, Concession model, Private information, Uncertainty, Relatedness

## Abstract

**Abstract:**

Understanding variation in reproductive skew between and within cooperatively breeding species is a key aim of social evolution. However, tests of reproductive skew models give equivocal results, potentially because different models make different assumptions and some of the theoretical assumptions are wrong. Most models assume that both dominants and subordinates are perfectly informed, but animals likely have asymmetric imperfect information, since individuals know better their own quality and subordinates are freer to explore breeding options outside the group. To explore effects of dominants’ uncertainty, we extended the standard concession model of skew with an explicit focus on subordinate quality, which we assume determines their outside options and influences their contribution to group productivity. Depending on how quality influences group productivity, dominants should prefer low- or high-quality subordinates. When subordinate quality correlates positively and strongly with group productivity, skew decreases with quality, otherwise skew increases with quality. The average concession offered to subordinates is greatest when dominants have imperfect information. In most cases dominants are selected to acquire information, whereas subordinates should restrict the information given to dominants, even though this may reduce the opportunities for cooperative breeding. Concessions always decrease with relatedness, so related subordinates would especially benefit from the dominant being uncertain about relatedness, potentially explaining why true kin recognition is rare in nature. Overall, our new predictions show that uncertainty can strongly influence evolutionary games and that incorporating it in skew models may to help explain patterns of cooperative breeding observed between and within species.

**Significance statement:**

Great variation in reproductive skew exists among cooperative breeders, which current theory fails to predict. This suggests the need for more realistic models. The level of uncertainty about the fitness consequences of the decision to breed cooperatively or not can vary between dominants and subordinates, and among dominants of different species. Our theoretical study predicts that a dominant’s level of information about the quality of a potential subordinate influences the optimal reproductive share and inclusive fitness. Furthermore, our work reveals that the link between a subordinate’s outside options and their effect on group fecundity is extremely important in shaping reproductive decisions and information-related strategies. Related subordinates should conceal their kinship to the dominant, potentially preventing the evolution of kin recognition. We argue that empirical studies should strive to disentangle the various ways in which subordinates affect fitness, and examine the variation among individuals in their opportunities and abilities to help.

**Supplementary information:**

The online version contains supplementary material available at 10.1007/s00265-025-03604-5.

## Introduction

In cooperative breeders, subordinates help to raise the offspring of dominant individuals. Whilst in some species dominants monopolise reproduction (Montague and Oldroyd 1998; Burland et al. 2002; Thorne et al. 2003; Liao et al. 2015), in many others reproductive skew (i.e., reproductive inequality) is not absolute because subordinates also reproduce, albeit at a lower level than dominants (Clutton‐Brock et al. 2010; Raihani and Clutton-Brock 2010; Kingma et al. 2011a; Lucas et al. 2011; Jaatinen et al. 2012; Hellmann et al. 2015; Andrade et al. 2016). The total productivity (e.g. number of offspring) that individuals achieve when in a group cannot be lower than the total productivity that individuals would achieve when breeding independently, because otherwise group members would get higher inclusive fitness if both bred independently. The efforts of a group are complementary in some way that increases group productivity to higher than their sum when alone. Yet, dominants and subordinates will usually disagree over the sharing of reproduction.

There are three broad classes of reproductive skew models: concession, restraint and tug-of-war models. In ‘transactional’ models—concession and restraint models—the reproductive share of the subordinate is a reward for the help that it gives to the dominant (Vehrencamp 1983); these models assume that individuals might leave the group. In ‘concession’ transactional models, the dominant has full control over the reproductive share and “pays” the subordinate to stay (Field and Cant 2009). Concession models predict that the reproductive share of subordinates should decrease (i.e., skew should increase) with the benefits that they bring to group productivity and their relatedness to the dominants (Vehrencamp 1983). In ‘restraint’ transactional models, subordinates restrain their breeding to avoid eviction by the dominant. Opposite to the concession models, restraint models predict that skew should decrease when subordinates bring greater benefits to group productivity and are more related to the dominants (Reeve et al. 1993). The tug-of-war models predict no correlation or a negative correlation between relatedness and skew (Reeve et al. 1998; Langer et al. 2004). The synthetic model provides a useful framework that reconciles the three main models of reproductive skew (Johnstone 2000). In this, the concession and restraint models define the minimum and maximum reproductive shares, respectively, while the tug-of-war determines skew within these two limits.

One factor that can influence the level of reproductive sharing is the likelihood that subordinates can breed successfully outside the group if they leave, referred to as their “outside option” (Creel and Rabenold 1994; Grinsted and Field 2017a,b). Outside options can be linked to size, rank, age, microsatellite heterozygosity and/or other parameters (Creel and Rabenold 1994; Heg and Taborsky 2010; Grinsted and Field 2017a). Outside options are likely to have an important influence on the decisions of subordinates to stay in or leave the group, and therefore on the dominant’s decisions too. For instance, in the concession model, increasing the fitness value of a subordinate’s outside options increases the reproductive concession that the dominant must offer to persuade the subordinate to stay and help (Johnstone 2000). In the restraint model, increasing the fitness value of a subordinate’s outside options increases the minimal share of reproduction the dominant will need to benefit from the subordinate staying in the group. In contrast to transactional models, the original ‘tug-of-war’ model (Reeve et al. 1998) assumes that individuals compete and cannot leave the group, so outside options are not directly considered. Extensions of the tug-of-war models have considered outside options by including the possibility for subordinates to leave the group to breed alone (Nonacs 2007,2010).

Empirical tests of these three types of models give inconsistent and equivocal results (Olivier, 2023). Most findings are consistent with the tug-of-war model in that, within species, skew does not correlate with relatedness nor group productivity (Nonacs and Hager 2011; Kaiser et al. 2018, 2019a; Oi et al. 2021; but see Lu et al. 2012; Miyazaki et al. 2014). Yet, the support for the tug-of-war model often relies on the absence of correlation between skew and a potential driver of skew, which may stem from more complex interactions than the ones tested or from studies not capturing a small effect. Furthermore, studies of some taxa support the predictions of both transactional and tug-of-war models—for example, work on European badgers *Meles meles* (Dugdale et al. 2008), hover wasps *Parischnogaster mellyi* (Fanelli et al. 2005, 2008) and myrmicine ants *Leptothorax acervorum* (Hammond et al. 2006)—which makes it difficult to know which model (if any) applies for each species. The conflicting predictions of the three models and the ambiguous empirical results prompt the need for a theory of reproductive skew that is biologically *richer* (sensu McNamara 2013), in at least two ways: by incorporating variation among subordinate individuals in their outside options and contributions to group productivity, and uncertainty about that variation and their relatedness to the dominant.

First, whilst the original models of skew assume that the group productivity is a constant (Vehrencamp 1983; Reeve and Ratnieks 1993), there is substantial intraspecific variation in the helping ability and effort of subordinates (Bergmüller et al. 2010; English et al. 2010; Zöttl et al. 2013; Green et al. 2016). Individual quality affects both this contribution of subordinates to group productivity and their outside options (Kokko and Johnstone 1999; Ragsdale 1999; Cant and Field 2001; Grinsted and Field 2017b; Koenig and Dickinson^2016^; van Boheemen et al. 2019). For example, subordinate genetic quality correlated positively with breeding pair reproductive success and offspring body condition in El Oro parakeets *Pyrrhura orcesi* (Klauke et al. 2013), which suggests that better-quality subordinates can contribute more to group productivity. Theoretical developments are needed to assess how the link between the subordinate’s outside options and their effect on group productivity might influence reproductive strategies. An agent-based simulation model by Nonacs (2019) implicitly explored how changing subordinate outside options and their helping effects influences skew, and suggested that varying the helping effects did not influence skew. In the simulation, when subordinates could choose among dominants with various levels of control over skew, the degree of skew increased with nest competition. Subordinate competitiveness increased with a reduction in nest availability, for all values of the helping effect (Nonacs 2019). This suggests that an increase in subordinate competitiveness is driven by the difficulty of founding their own nest (i.e., high breeding site competition). By contrast, dominants’ competitiveness changed little with nest availability, because dominants already have a guaranteed spot to breed. Nonacs’s (2019) simulation model highlighted the importance of variation in cooperative breeders’ decisions. However, his simulation did not investigate how the link between subordinate outside options and their effect on group productivity changes skew. In the study presented here, we develop an analytical model that explicitly formulates this link.

The second limitation of existing models of skew is that they typically assume perfect information, in that all individuals can base their decisions on all influential factors such as the subordinate’s outside options and its relatedness to the dominant (Vehrencamp 1983; Reeve and Ratnieks 1993; Johnstone 2000; Kokko and Ekman 2002). In reality, animals make decisions based on imperfect information, because their social and abiotic environments vary and they cannot be omniscient (Dall et al. 2005). How information about relatedness or subordinate quality influences reproductive skew has been little explored, despite some indications that incorporating uncertainty will affect predictions. For example, Kokko (2003) showed that when subordinates do not know perfectly the benefits of staying in the group, invasion by cheating dominants that concede nothing undermines the stability of cooperative breeding. Similarly, limited information about one another’s outside options can prevent cooperative breeding even when forming a group would be mutually beneficial, as shown in a model by Akçay et al. (2012). These predictions suggest that the current models do not sufficiently capture the key factors that determine cooperative breeding, given that some animals evidently do achieve cooperation despite imperfect information. Because imperfect information can cause sub-optimal strategies compared to the games where information is assumed to be perfect, models that integrate uncertainty about a subordinate’s outside options and relatedness will improve our understanding of reproductive skew and cooperative breeding.

In Akçay et al.’s (2012) model, roles were asymmetrical: when forming a group, one individual gave up a share of reproduction (similar to a dominant) while the other gained a share of reproduction (similar to a subordinate). The uncertainty was symmetrical in that both individuals had limited information about the outside options of the potential partner (Akçay et al. 2012). But instead of symmetric private information, it is likely that subordinates have more information than dominants about subordinates’ outside options. First, individuals typically know more about their own abilities (e.g., body condition, size, strength) than they do about those of others (Bridge et al. 2000; Arnott and Elwood 2009). Second, the outside option (i.e., independent breeding success) of an individual is a combination of both its ability and the quality of the (potential) breeding sites. A dominant’s outside option is assumed here to be solitary breeding in their current nest, whereas a subordinate’s outside option entails breeding elsewhere. Both dominants and subordinates have direct experience of the breeding-site quality of the dominant, and therefore have high information about the dominant’s outside option (Barve et al. 2020). Subordinates, on the other hand, may make external forays to explore different outside breeding options (Young et al. 2007) and assess the qualities of these outside breeding options, which is largely private information because the dominant cannot know which particular breeding site the subordinate favours. Consequently, dominants have higher uncertainty about the subordinate’s outside option than the subordinate. Third, variation in the outside option may be much smaller for dominants than for subordinates, because individuals need to reach a threshold of quality (e.g., dispersal age) to become dominant and are then constrained by solitary breeding ability and limited resources at the breeding site (Creel and Rabenold 1994; Stephens et al. 2005 but see Boyd 1992).

Cooperative breeders are commonly monogamous (Hughes et al. 2008; Cornwallis et al. 2010; Lukas and Clutton-Brock 2012), but there is great diversity in kin structure (Koenig and Dickinson 2016). Some groups accept non-natal helpers and extra-pair paternity does occur, making help not exclusively kin-directed (Cockburn 1998; Clutton-Brock et al. 2002; Kingma et al. 2011b; Kaiser et al. 2019). Skew models have tended to assume perfect information about the relatedness between dominants and subordinates (Kokko et al. 2001; Komdeur et al. 2008; Holman 2014; Kuijper and Johnstone 2019; Nonacs 2019). Concession models, for example, predict that dominants should concede less reproduction to more closely related subordinates (Hamilton 1964; Vehrencamp 1983). But dominants lacking perfect information about kinship may over- or underestimate how related they are to a subordinate, and therefore what concession to offer, which in turn has consequences for the subordinate’s decision to stay and help or leave and attempt to breed independently. Theoretical investigations of the effect of uncertainty, assessing it independently for each individual, would help to assess the importance of information about relatedness in the evolution of cooperative breeding and reproductive skew.

Depending on the species and the environmental conditions, having good outside options may correlate with high benefits to group productivity or with a low or negative effect on group productivity. For instance, helper quality is positively linked to young body condition in El Oro parakeets (Klauke et al. 2013). By contrast, subordinates with experimentally increased nesting and partner availability reduced their helping efforts in *Polistes dominula* paper wasps (Grinsted and Field 2017b), which likely reduces group productivity. We term this link between subordinate outside options and the benefit they provide to group productivity the “quality–productivity coefficient” (QPC). If a subordinate’s QPC is negative, having better outside options is associated with a lower helping effect, which could happen when individuals specialise in breeding or helping. Naked mole-rats *Heterocephalus glaber*, where subordinates show distinct helper and disperser morphs (O’Riain et al. 1996), would be an example of negative QPC. Negative QPC might also occur if breeding success is underpinned by a mechanism that counteracts prosocial behaviour (e.g., testosterone; Vernasco and Moore 2020). Therefore, subordinate quality (i.e., outside options) might have different effects depending on the QPC. Models of skew predict that subordinate quality should influence group formation, because it determines the fitness subordinates will get if breeding alone. Yet previous work has confounded subordinate quality and their QPC by studying only the helper effect on group productivity (Woxvold and Magrath 2005; Williams and Hale 2006; Doerr and Doerr 2007; Sparkman et al. 2011; Savage et al. 2015; Grinsted and Field 2018; Jacobs and Ausband 2019), thus failing to capture how variation among subordinates affects cooperative breeding decisions.

Here, we develop a model that incorporates these overlooked elements of biological richness, to paint a fuller picture of the reproductive decisions of cooperative breeders. Specifically, we examine the effect of varying the association between the subordinate outside option and its contribution to group productivity, and the dominant’s level of information about the subordinate. Since our focus was on the use and value of information, it was both convenient and clearer to base our work on a simple model rather than on a complex synthetic model. For similar reasons, we follow a long tradition in assuming that evolution has led to rules that individuals follow, non-flexibly. As a starting point, we based our model on the concession model of reproductive skew (Reeve and Ratnieks 1993), because this explicitly assumes that the subordinate has an outside option. The suitability of transactional models for predicting the share of reproduction has been questioned. Nonacs and Hager (2011) argue that reproduction is determined by an intrinsic quality of individuals that cannot be won, and that therefore it does not make sense to consider reproduction as divisible. Yet, the winner effect suggests that individuals who win a fight subsequently become more aggressive and are more likely to win a second fight (Wazlavek and Figler 1989; Morino 2016), which suggests that individual quality varies and depends on the context (e.g., possession of a breeding site). Besides, unless subordinates are sterile (e.g., honeybees), subordinates can attempt mating and/or lay eggs. Many species have multiple breeding attempts of multiple offspring, so reproduction is divisible (Fanelli et al. 2005). Dominants suppress subordinate reproduction physiologically or behaviourally in at least some vertebrates and invertebrates (Tibbetts et al. 2018; Creel 2022), but this hormonal or behavioural control is flexible and can change as soon as the subordinate becomes dominant or leaves the group to breed (Tibbetts and Izzo 2009). A dominant’s decision to evict subordinates depends on the costs that the subordinates’ reproduction imposes on the dominant and its offspring, but also on the benefits of keeping the subordinate (even though the subordinate reproduces). Therefore, the concession model can apply and we chose to extend this framework.

We extend the basic concession model by assuming that subordinate intrinsic quality is equivalent to their outside option, but that their contribution to group productivity may vary positively or negatively with the outside option. As such, we introduce variation in the QPC. We also examine the effect of uncertainty about the subordinate’s relatedness to the dominant, and about the former’s outside options. The model by Akçay et al. (2012) also focuses on information about the other’s outside options, but here we explicitly assume that dominants have less information than subordinates: subordinates know perfectly the outside options of the dominant (i.e., solitary breeding success estimated from breeding-site quality), but not vice versa, because subordinates explore and collect more information about alternative breeding opportunities elsewhere.

## The model

We extended the transactional concession model of reproductive skew (Reeve and Ratnieks 1993) by incorporating variation in subordinate quality (first and second sections) and relatedness (third section). Subordinate quality (*x*) affects both their outside option and group productivity (Table 1), and is equal to the subordinate’s fitness payoff associated with the outside options—biologically, this can depend on the environment but also on the breeding potential of the individual (low quality can reflect sexual immaturity or sterility). In our model, subordinate quality does not refer to the abilities of the subordinate to engage in reproductive conflict (tug-of-war) or mount a dominance challenge, and is independent from within-group competition.

**Table 1:** Variables and parameters in the model and their baseline and explored values

Symbol		Description	Baseline value	Explored values (figures)
*Individual traits*				
*x*		Quality and outside option of the subordinate (i.e. direct fitness if breeding independently) $0<\ =\ \text{x}<\ =\text{b}$	0.5	0 – 1 (Fig. 1, 2, 3, 4, 6, A1, A2, A3)
*x*_*i*_		Quality of individual *i *	0.5	0 – 1
*x*_*c*_		Critical quality *x* above which subordinate will leave to breed alone	Solved	
*y*		Reproductive share offered to subordinate by dominant (i.e. proportion of group productivity)	Solved	0 – 1 (Fig. 1, 2, 3a-c, A4)
*y*_*c*_		Critical share above which subordinate will stay and help	Solved	
y*		Optimal reproductive share for range of subordinates	Solved	Equation A10 (Fig. 1a-d, 4a-c)
y*_P_		Optimal reproductive share when dominants have perfect information about *x*	Solved	Equation 1.9 (Fig. 1)
*z*		Perceived subordinate’s quality, when dominants have information ω	0.5	0 – 1 (Fig. 2, A1, A2)
*G*		Group productivity (fecundity per breeding season)	*G=b(1+h)*	Equation 1.5
*Group traits*				
*b*	Direct fitness of a solitary dominant breeder		1	(Fig. A4)
*h*	Effect of helping on group reproductive output		*h = ax + m*	Equation 1.4
*m*	Benefit of cooperation: Minimal effect of helping on group reproductive output (i.e.		*0.35*	Equation 1.4 (Fig. 1,5, A3)
*a*	Effect of quality *x *on group productivity: subordinate’s quality-productivity coefficient (QPC)		0.5	-1 – 2, Equation A16 (Fig. 1, 3, 4, 5, A4)
*d*_*I*_	Productivity of a dominant (i.e. direct fitness)			Equations 1.6, 1.8
*s*_*I*_	Productivity of a subordinate (i.e. direct fitness )			Equations 1.7
*Individual fitness*				
*d*_*P*_	Dominant inclusive fitness with no information (i.e. mean reproductive value of dominants)		Optimised	Equation 1.8 (Fig. 1, 3d-f)
*D*_*A*_	Dominant’s expected inclusive fitness for all subordinates			Equations 1.3, A3
*v*_*A*_	General inclusive fitness of solitary dominants			Equation C1 (Fig. 1, 4)
*v*_*P*_	General inclusive fitness of dominants with perfect information			(Fig. 1, 4)
*v*_*N*_	General inclusive fitness of uninformed dominants			Equation A23 (Fig. 1, 4)
*d*_*A*_	Dominant inclusive fitness when alone (i.e. mean reproductive value of solitary dominants per breeding season)			Equation 1.1
*d*_*D*_	Direct fitness of a dominant who breeds cooperatively			Equation A4
*s*_*D*_	Direct fitness of subordinate who breeds cooperatively			Equation A4
*d*_*C*_	Inclusive fitness of a dominant when cooperatively breeding			Equation A7
*s*_*P*_	Subordinate inclusive fitness when dominants have perfect information (i.e. mean reproductive value of subordinates per breeding season)			Equations A20, A40 (Fig. 1)
*s*_*N*_	Subordinate inclusive fitness when dominants have no information (i.e. mean reproductive value of subordinates per breeding season)			Equation A44
*r*	Symmetric relatedness between the dominant and subordinate		0.25	0 – 1 (Fig. 5, 6)
Information parameters				
ω	Dominant’s information about subordinate’s quality $0<\;=\omega\;<\;=\;+\infty$		2^4^= 16	0, 2^0^, 2^4^, 2^10^… ∞ (Fig. 2, 3, 4)
*P(z|x)*	Probability that quality *x *is perceived as quality *z.*			Equations 1.10, A52
*α, β*	Parameters of the beta probability distribution *P(z|x)*			Equations 1.10, A50
*Ω*	Dominant’s information about relatedness to the subordinate		2^4^ = 16	0, 2^0^, 2^4^, 2^10^… ∞ (Fig. 5, A4)
*θ*	Beta-weighted distribution of what dominant infers from observation ωabout* x*			Equation A51
*s*	Mean reproductive value of the subordinate		Optimised	(Fig. 1, 3g-i)

Following the tradition of basic skew models, we only consider the decision to breed alone or with one other individual (cooperative breeding occurs when a dominant and subordinate form a group); i.e. 2-player game. We do this because information has not much been understood, and it remains for future work to develop a N-player game. A priori, like in previous skew models (Johnstone 2000), dominants and subordinates are fundamentally different. Indeed, in many social groups, the subordinate helpers are younger individuals (e.g. immatures helping their families: Hagen and Barrett 2009), reproductively suppressed (gerbils: Saltzman et al. 2006, carnivores: Montgomery et al. 2018), or completely sterile individuals (eusocial insects e.g. Montague and Oldroyd 1998). At least, there is a hierarchical difference which presupposes a difference in competitive ability or that the dominant was natal to the breeding site (contrary to the subordinate) (Fawcett and Johnstone 2010). Subordinate contribution to group productivity (hereafter “helping”) may vary positively or negatively with the outside option. Individuals are fixed in their behaviour. For instance, helping is fixed, not conditional, as we consider a one-off interaction where individuals make a simultaneous decision and cannot respond to each other. We focus on the decision made during one breeding season, rather than lifetime fitness strategies, therefore queuing and breeding site inheritance are not considered in the fitness calculations. Relatedness between subordinate and dominant (*r*) is symmetrical. Although our model aims to be generalisable to many cooperative breeders, we had in mind dwarf mongooses (Arbon et al. 2024), banded mongooses (Mitchell et al. 2018), meerkats (Clutton-Brock et al. 2010) and various cooperatively breeding birds as model species whilst building this model (e.g. Rabenold 1985; Woxvold and Magrath 2005; Williams and Hale 2006; Kingma et al. 2011; Riehl 2017; Kaiser et al. 2018, 2019; van Boheemen et al. 2019).

The contributions to the inclusive fitness of a dominant (*d*_*A*_) and subordinate (*s*_*A*_) that share a proportion *r* of their genes by common descent if they do not cooperate (i.e. subordinate leaves or is evicted) are

1.1 $$d_{A} = b + rx,{\text{and}}$$

1.2 $$s_{A} = rb + x,{\text{ respectively}}.$$

Without loss of generality (because all else scales), we assume that subordinate quality is uniformly distributed between 0 and 1, and that dominants have quality equal to the highest-quality subordinate (*b* = 1). Note that in our model, the dominant’s relative competitive ability against the subordinate *b* (1 − subordinate efficiency) is different from subordinate quality* x*, although we do assume that the maximum subordinate quality is *b*. We did not assume that the subordinate’s productive ability (*x*) equals its fighting ability (competitive ability against the dominant); this assumption would have implied that a subordinate with maximum quality *b* has a 50% chance of gaining the dominant position.

The dominant’s expected fitness for all subordinates is therefore

1.3 $$D_{A} = b + \frac{r}{2}$$

## Comparing perfect information and no information about subordinate quality

### Methods: comparing perfect information and no information about subordinate quality

We start from the baseline case in which the dominant has perfect information about subordinate quality *x* to explore the impact of the subordinate’s QPC (denoted *a*) on the basic concession model. The subordinate provides a benefit *h* to the group as a helper*,* which increases linearly with its quality *x,* with slope *a* and intercept *m* (Table 1, Eq. 1.4).

1.4 $$h = ax + m$$

If *a* < 0, subordinates with good outside options will be poorer helpers, whereas if *a* > 0 they are better helpers. The additional group productivity from the subordinate staying is assumed to be the product of *h* and the dominant’s quality *b*; thus, the total group productivity *G* is

1.5 $$G = b + bh = b(1 + m + ax)$$

Hence, if the subordinate stays to help, the direct fitness of a dominant who gives reproductive concession *y*_*i*_ is

1.6 $${d}_{f}=\left(1-{y}_{i}\right)G=b\left(1-{y}_{i}\right)\left(1+m+ax\right)$$

and that of the subordinate is

1.7 $${s}_{I}={y}_{i}G={by}_{i}\left(1+m+ax\right)$$

So the dominant’s inclusive fitness is

1.8 $$\begin{array}{c} d_{P} = d_{I} + rs_{I} = b(1 - y_{i} )(1 + m + ax) + bry_{i} (1 + m + ax) \\ d_{P} = b[1 - y_{i} (1 - r)](1 + m + ax) \end{array}$$

For* r* < 1, the dominant’s fitness decreases as *y*_*i*_ increases, so they should give the smallest concession that will induce the subordinate to stay, which by rearrangement of (1.8) is

1.9 $$y_{P}^{*} = \frac{x - br(m + ax)}{{b(1 - r)(m + ax + 1)}}$$

We can get analytical results for the perfect and no-information extremes (Appendix A).

**Fig. 1:**
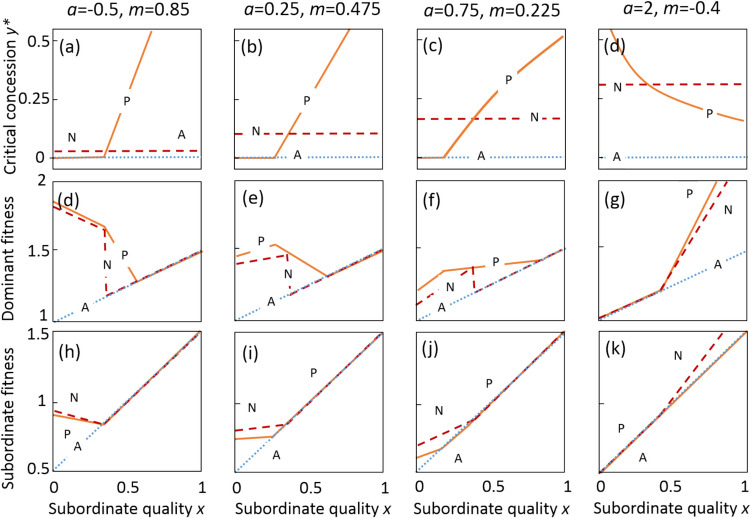
Optimal concession (**a**-**d**), dominant inclusive fitness (**e**–**h**) and subordinate inclusive fitness (**i**-**k**) for some representative values of *a* and *m* (columns) under perfect information *P*, no information *N*, and when alone *A*. The dotted lines in (**a**-**d**) indicate *y* = 0. The dominant fitness depends on whether the dominant wants the subordinate to stay (*d*_*P*_, *d*_*N*_ > *d*_*A*_) and whether the subordinate chooses to stay (*d*_*N*_*, s*_*N*_ > *s*_*A*_). The dominant always does at least as well as no information when they have perfect information, whereas the opposite is true of the subordinate. Other parameter values: *r* = 0.5, *b* = 1

First, to understand the broad effect of information, we explored the interaction between the quality–productivity coefficient *a* and subordinate quality *x* in their effect on the optimal concession and fitness outcomes when the dominant has either perfect or no information. We could get analytical results for the effects of this absence of information (Fig. 1, Table 2). We set the dominant’s solitary breeding success at *b* = 1 throughout, to be equal to the highest-quality subordinate. To study the effect of *a* on the slope of fitness and the optimal concession, while avoiding changing the magnitude of the fitness with *a*, we kept *h* constant for the average subordinate (*x* = 0.5) by negatively linking *m* to *a* following *m* = 0.6 − *a*/2, so that the total group productivity for the average quality was always 1.6 (Fig. 1, Eq. 1.4). Thus, the expected group productivity for the average subordinate is slightly higher than the sum of the solitary breeding productivities (*b* + *x* = 1.5).

**Table 2:** Predicted effects of subordinate’s quality-productivity coefficient (QPC, *a*) on the directions or amounts of the reproductive strategies of cooperative breeders

Effect	Subordinate’s quality-productivity coefficient* a*	
Behaviour	Low (*a* < 1)	High (*a* > 1)
x on y*	positive	negative
Optimal concession y* (how does it vary with information about x?)	y*Perfect lower than y*NoInfo; y* maximal at low information	y*Perfect higher than y*NoInfo y* maximal at No Information
Optimal concession y* with r	decrease	decrease
Cooperative breeding	low *x*	high *x*
Solitary breeding	high *x*	low *x*

Optimal concession y* is the reproductive share that maximises inclusive fitness

### Results: comparing perfect information and no information about subordinate quality

We show that having no information about subordinate quality affects the predictions of the basic concession model (Fig. 1). The analysis predicts that when dominants have perfect information, concession should increase with subordinate quality *x* (Fig. 1a–c) unless the effect of *x* on group productivity is very strongly positive (*a* > 1, Fig. 1d), in which case higher-quality subordinates are willing to stay regardless of concession, so dominants can offer less. Dominants should breed cooperatively with low-quality subordinates when *a* is weak or negative (*a* < 0.6, Fig. 1e, f) because for low-quality subordinates (but not high-quality ones), dominant fitness is higher with the subordinate than alone. However, as for strong positive *a*, retaining a high-quality (but not a low-quality) subordinate increases dominant fitness compared to solitary breeding, so dominants should breed cooperatively with high-quality subordinates (Fig. 1g, h). These effects occurred when dominants had perfect and no information. Unexpectedly, the effect of *x* on payoffs and skew for average subordinates (around *x* = 0.5) is negative if *a* is small or negative, but positive if *a* is large (Fig. 1a-d, j-l).

Dominants with no information get a lower payoff than those with perfect information across much of the range of *x,* but most strongly for intermediate values where they fail to breed cooperatively when they should (Fig. 1e-h). Subordinates get higher inclusive fitness as a subordinate when *x* and *a* are either both high or both low (Fig. 1i–l) because dominants offer more than they need to induce the subordinate to stay.

## Uncertainty about subordinate quality

### Methods for imperfect information about subordinate quality

In between the extreme cases of perfect and no information, we use Bayes’ theorem in a numerical model to explore the effect of increasingly accurate information on the decisions and inclusive fitness of both individuals. We assumed that information affects the distribution of possible subordinate qualities considered by the dominant, for a given true quality *x*. In brief (see Appendix A for details), given an actual quality *x*, the probability distribution of the dominant’s perception of this quality, *z*, follows a beta distribution

1.10 $$P(z\vert x)=\frac{z^\alpha(1-z)^\beta}{B(\alpha,\;\beta)}\\$$

where *B*(*α*,*β*) is the sum of the distribution in [0,1] and the error is controlled by the amount of information *ω*, with

1.11 $$\begin{aligned} \alpha & = 1 + x\omega l \\ \beta & = 1 + (1 - x)\omega \end{aligned}$$

such that higher values of *ω* give a narrower distribution. Note that if *ω* = 0 (no information), all *z* (0 ≤ *z* ≤ 1) are equally likely, and if *ω* = ∞ (perfect information) then P(*z*|*x*) = 1 if *z* = *x*. The dominant should make their decision based on the probability of each actual quality *x* given its perception *z*, which we calculate using Bayes’ rule:

1.12 $$P(x\vert z)=\frac{P(z\vert x)\cdot P(x)}{P(z)}$$

The dominant finds the optimal concession given the inclusive fitness consequences for each *x*, which will influence whether the subordinate leaves or stays, weighted by *P*(*x*|*z*). Dominants and subordinates may be in conflict about the quantity of information (*ω*) that the dominant has about *x*. After finding the optimal concession y*, we find the proportion of the population of subordinates for which the dominant and subordinate would choose to stay in the group and the fitness consequences for each *x* by using the weighting $P(x|z)P(x)$.

In the baseline concession model, relatedness is 0.25 to mimic the common situation where subordinates help half-siblings or cousins (Rabenold 1985; Härdling et al. 2003). We chose 0.25 as “average”, which incorporates family-based cooperation to coalition-based polygyny; this value reflects the fact that although the majority of cooperative breeding occurs in family groups (Hatchwell 2009; Rosenbaum and Gettler 2018), subordinates do not always help raise full-siblings (due to divorce, one parent dying, extra-pair copulation) and in some species subordinates help non-relatives (e.g. banded mongooses: Marshall et al. 2021). In dwarf mongooses, where the dominant female gives birth to nearly 90% of the pups, subordinate females and males are only related to the same-sex dominant by *r* = 0.31 and *r* = 0.27 respectively (Arbon et al. 2024).

We developed a similar numerical model to explore how the dominant’s information about their relatedness (*Ω*) to the subordinate influenced the predictions (see details in Appendix B). We considered relatedness values within the range [0–1]. Uncertainty about the subordinate’s relatedness was expected to influence the reproductive decisions, because the dominant would not be able to compare accurately its expected inclusive fitness when breeding cooperatively and alone. Note that we show results for all possible *z*, but for *ω* > 0 not all *z* are equally likely. Thus, calculating fitness outcomes must take the distribution of (*x,z*) into account.

### Results for imperfect information about subordinate quality

#### Responses to perceived subordinate quality

**Fig. 2:**
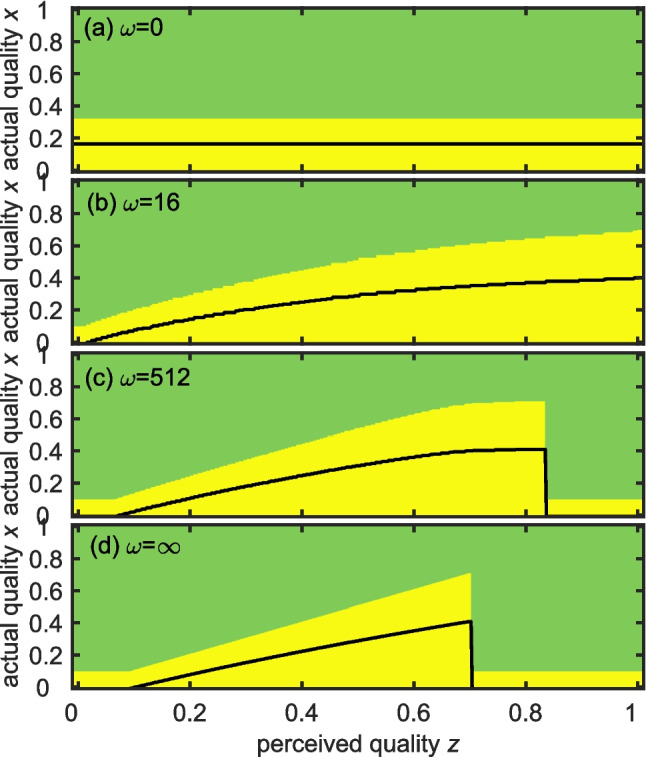
Effect of perceived quality of subordinate *z* on the optimal concession y* (black lines) for four levels of knowledge: **a** no knowledge ω = 0, **b** some knowledge ω = 16, **c** high information ω = 512, **d** perfect information ω = ∞. The colours show the areas of perceived quality by dominant (horizontal axis) and actual quality of subordinate (vertical axis) where for the optimal *y* both dominant and subordinate would do better in a group (green)*;* only the dominant would do better in a group (yellow). Other parameter values: *a* = 0.5, *m* = 0.35, *r* = 0.25

To understand the role of imperfect information, we explored group membership decisions given the dominant’s optimal concession *y*_*P*_(*z*) for the range of perceived (*z*) and actual (*x*) quality: whether the subordinate wants to stay and whether the dominant wants them to stay (Fig. 2, Fig. A3; see Appendix A for details). For moderate effects of subordinate quality and moderate relatedness (i.e. *a* = 0.5, *m* = 0.35, *r* = 0.25), the dominant always wants the subordinate to stay given *y*_*M*_ (*z*) (Fig. 2), but the subordinate does not stay if the concession is too low.

**Fig. 3:**
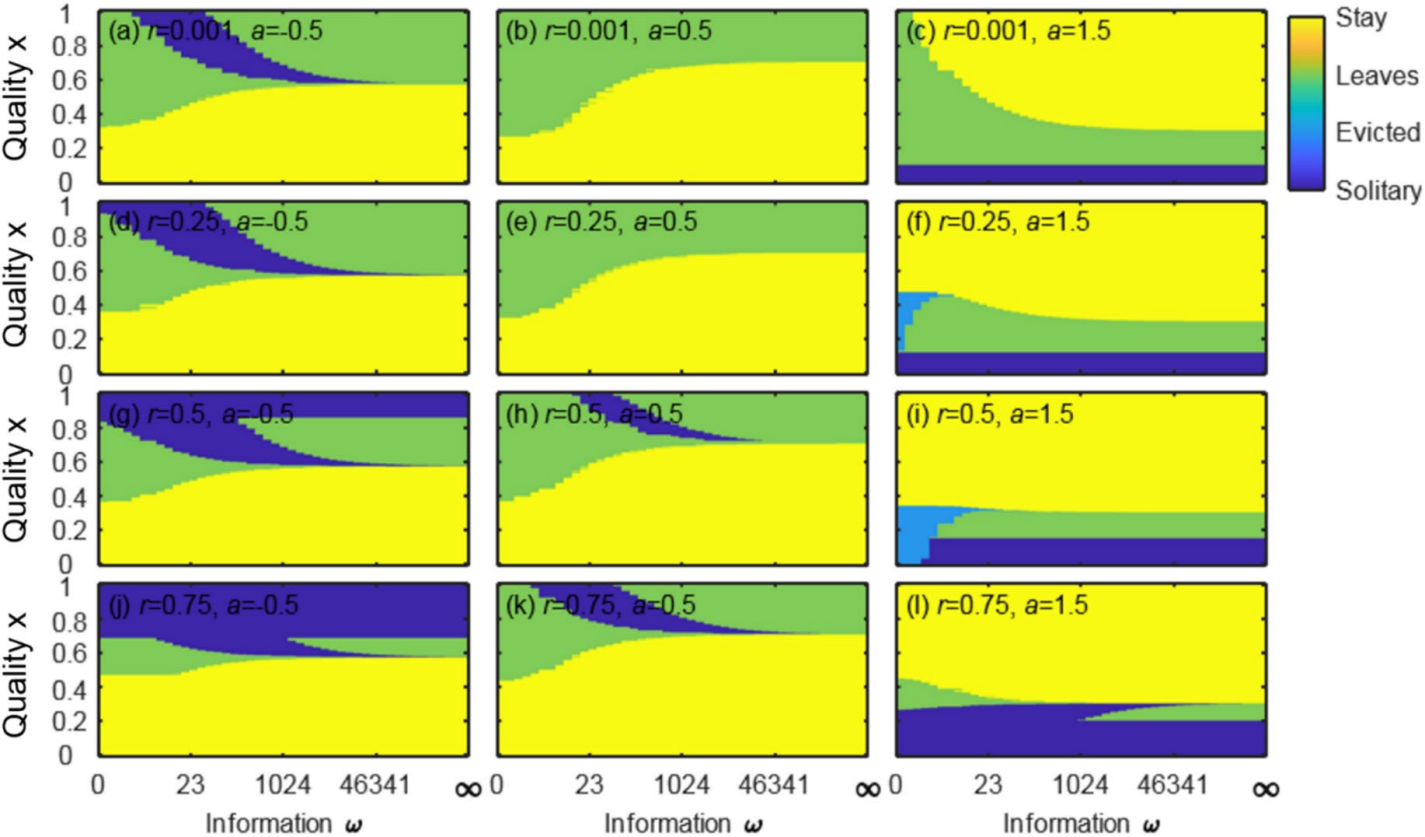
Effect of information about *x* on the prediction of cooperative breeding for various QPC (*a*) and relatedness (*r*). The colours show the areas of dominants’ level of information about *x* (horizontal axis) and actual quality of subordinate (vertical axis) where the average decision across all perceived subordinate qualities was: to stay for both dominant and subordinate (green); only the dominant wants to breed cooperatively (yellow); only the subordinate wants to breed cooperatively (cyan); or neither wants to breed cooperatively (blue), for 3 values of quality-productivity coefficient *a* (columns) and four values of relatedness (rows); values shown on panels

In all cases, the range of actual subordinate qualities where groups fail to form increases as information decreases (height of yellow areas decreases right to left). Under no information (Fig. 2a), y* is constant (since there is no perceived quality *z*) and subordinates stay only if their actual quality is below a constant threshold. With *ω* > 0, y* increases with *z* and so this threshold also increases (Fig. 2b-d). For good or perfect information (Fig. 2c, d), there is a *z* above which the dominant perceives the necessary concession to be too great, so they offer nothing, but very low-quality subordinates would still stay due to the indirect fitness benefits of helping.

**Fig. 4:**
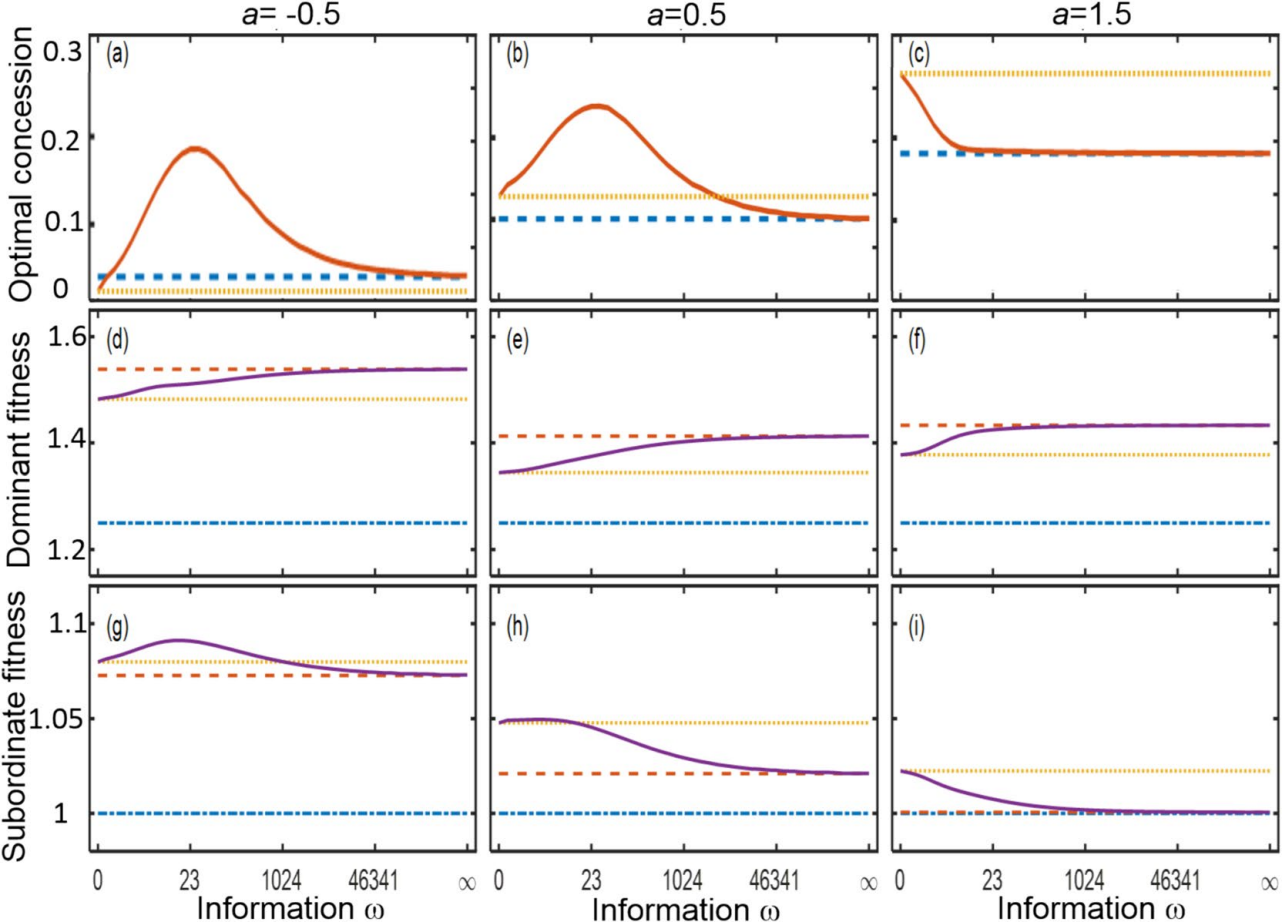
Effect of information about *x* on decisions and fitness outcomes for all possible subordinates. **a**-**c** optimal concession (y*); **d**-**f** average dominant inclusive fitness and (**g**-**i**) average subordinate inclusive fitness, as a function of the quantity of information about the subordinate’s outside option. Columns show different values of quality-productivity coefficient and synergy effect *(a, m)* (columns: left *a* = − 0.5, *m* = 0.85; middle *a* = 0.5, *m* = 0.35; right *a* = 1.5, *m* = − 0.25). Lines indicate gradually increasing information (solid line), and analytical solutions for perfect information (dash line), no information (dotted line), fitness alone (dot-dash line). Other parameter values: *b* = 1, *r* = 0.5

The quality–productivity coefficient (*a*) strongly influences group formation. Dominants with no or imperfect information always would do better with a subordinate if *a* = 0.5 (Fig. 2), but not for high perceived quality if *a* = − 0.5 or 0.25 (see in Appendix A Fig. A3). When *a* is low but positive, dominants prefer to form a group but subordinates prefer to breed alone if the concession is not higher than their quality *x*, as it is not sufficient to retain them (Fig. 2). When *a* < 1, dominants with low information gave the highest concession (Fig. 3a, b), due to the shape of the relationship between the (expected) quality and concession (Fig. 2). When *a* is low (Fig. 4 left and middle), there is a threshold of minimal subordinate quality below which groups are unlikely to form because on average subordinates prefer to breed alone (green and blue areas). When the effect of subordinate quality on group productivity is negative (left column), the regions of solitary breeding are larger because the group productivity is smaller. When group productivity strongly depends on subordinate quality (i.e. high *a*; Fig. 4 right), dominants would breed cooperatively with subordinates of high, but not zero, quality (Fig. 4c, f, i) and the threshold of minimal subordinate quality decreases with relatedness.

For higher relatedness (Fig. 4h, k), there is a region of moderate information where both dominants and subordinates prefer to breed alone, because breeding separately increases the dominant’s inclusive fitness compared to the large concession the subordinate would require to stay. There is a region of low quality and low information where moderately related subordinates want to stay, but the dominant does not want them because they would reduce group productivity to a potentially large degree.

#### Effect of uncertainty about subordinate quality on mean fitness

When group productivity moderately increases with subordinate quality (*a* = 0.5, Fig. 4 middle column), dominants with perfect information give a smaller concession than those with no information, but the effect of information on concession size is not monotonic, with a maximum at some intermediate information level (Fig. 4b). This occurs because having some information may allow the dominant to know that subordinates are not very low-quality ones who should be offered zero because they only moderately increase productivity. Therefore, greater concessions can be given knowing that the investment will be worth it. At higher information, dominants know that the subordinate is not high quality so can offer only what is necessary. Intuitively, dominant inclusive fitness increases with information (Fig. 4e) but with diminishing returns as concessions and payoffs are similar for subordinates of similar quality. Due to the effect on concession, the effect of information on subordinate fitness is not monotonic (Fig. 4h) but is always lowest when dominants have perfect information.

A decreasing effect of *x* on group productivity (*a* < 0, Fig. 4 left column) has similar results to when 0 < *a* < 1. On the other hand, when *x* strongly positively affects group productivity (*a* > 1, Fig. 4 right column), information always decreases the concession and so subordinates have highest fitness when dominants have no information.

#### Comparison to previous work on information and skew

To compare our results to those of Akçay et al. (2012), where helping effects do not vary with individuals’ outside options, we set a constant group productivity (*a* = 0, *m* = 1/*b* − 1) and altered the qualities of both the dominant (*b*) and the subordinate (*x*) (for details see Fig. A4). Cooperative breeding occurs when subordinate quality is high and dominant quality is low, since then the dominant benefits most and is willing to give a large concession (Fig. A4a, d). Uncertainty creates a large region of space in which the dominant would accept the subordinate but does not give a sufficiently large concession, and this area is partly in the space where groups would form under perfect information. Between these two regions of consensus where both either want to form a group or breed alone, dominants fail to retain subordinates (Fig. A4a-c). The zone of conflict over group formation is where the benefit of cooperation *m* and the subordinate’s outside option *x* add up to a small positive value. Thus, the results of Akçay et al. (2012) are robust to departures from the assumption of symmetrical information.

#### Optimal information about subordinate’s quality

Our model shows that information about the quality of a potential subordinate, as opposed to mere information about the probability distribution of this quality, increases dominants’ inclusive fitness. However, dominant fitness does not increase linearly but is asymptotic (Fig. 4d-f); therefore, if there are costs or constraints of acquiring or using information, then selection would not lead to perfect information. For illustration, we assume each unit increase in information *ω* costs an arbitrary 0.003 units of payoff, which allows us to find where the effect of gaining information is negligible; we refer to this as the ‘optimal’ information for dominants. By contrast, increasing dominant information has a non-monotonic effect on fitness for most subordinates in most situations (Fig. 4h-i), so no costs are assumed when seeking the optimal information for subordinates.

**Fig. 5:**
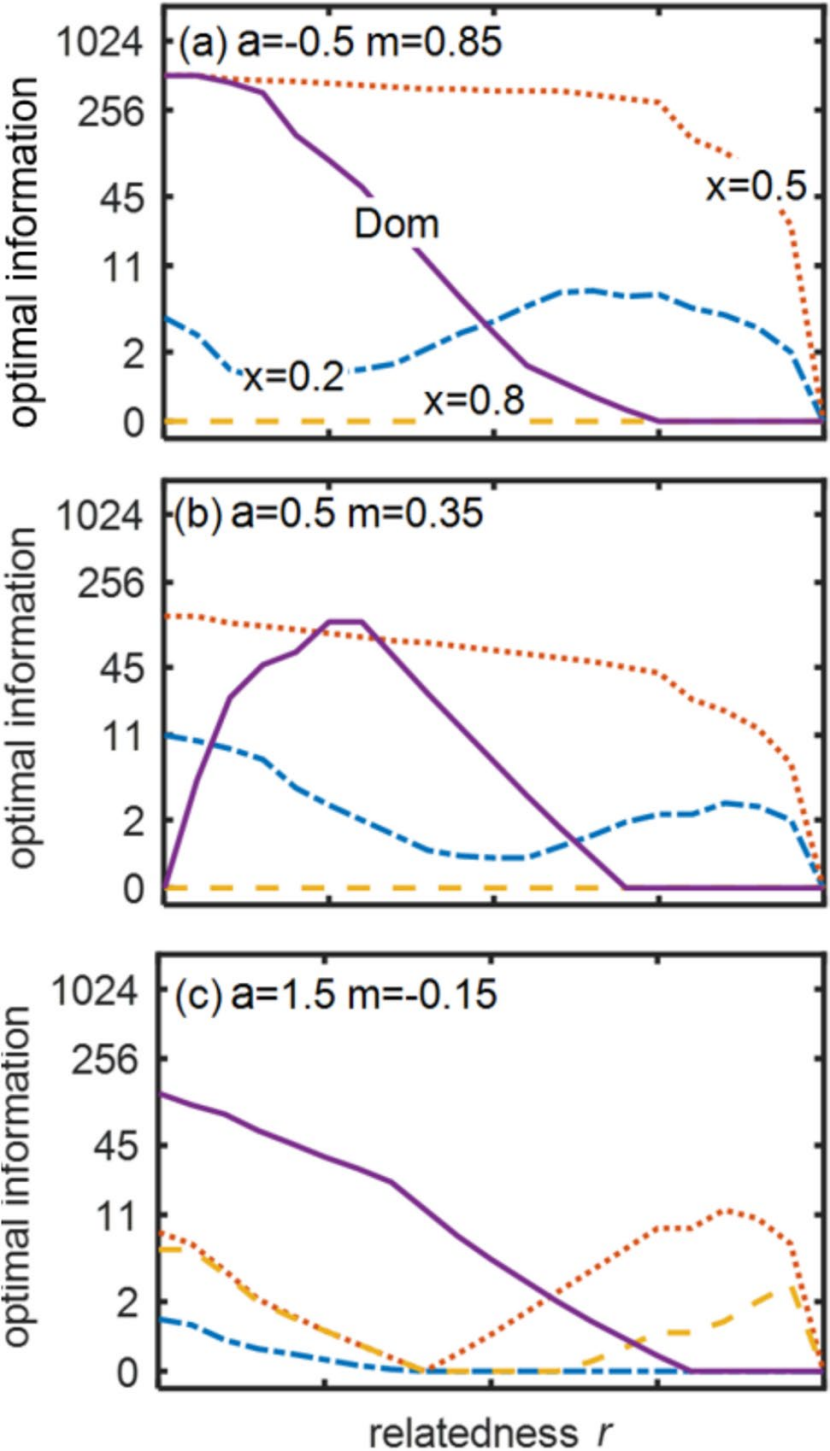
Optimal amount of information about quality for dominants and subordinates of three different qualities (lines) for three values of the quality-productivity coefficient *a* (rows). Lines indicate dominant (solid lines), low-quality subordinate (*x* = 0.2, dot-dash lines), average subordinate (*x* = 0.5, dotted lines), high-quality subordinate (*x* = 0.8, dashed lines)

In general, the optimal information for dominants decreases as relatedness increases (Fig. 5, solid lines), because necessary concessions are lower and have less effect on the dominant’s fitness due to indirect fitness when the subordinate breeds. The exception is where *a* = 0.5 and relatedness is low (left of Fig. 5b), when increasing relatedness increases the need for information because low-quality subordinates will stay even if they get zero concession, so it is worth identifying these subordinates.

The optimal information is very different for different qualities of subordinate, being in general greater for average-quality subordinates (Fig. 5, dotted lines) because the optimal concession is greatest at intermediate *x*, so these subordinates want to be distinguishable from the others. This declines as relatedness increases because the concession approaches zero. The highest-quality subordinates (Fig. 5, dashed lines) rarely get to stay, so it is better for them if dominants have less information, as then they will offer some concession.

## Uncertainty about relatedness

### Methods for imperfect information about relatedness

Individuals may have uncertainty not only about quality, but also about relatedness. We ran similar analyses to those above but with *x* known perfectly to the dominant and varying and uncertain *r*. See Appendix B for details.

### Results for imperfect information about relatedness

For all feasible values of parameters, the optimal concession decreases as relatedness and information increases (see in Appendix B Fig. B1). The magnitude of this varies with *x* and *a.* Consider a full offspring or sibling (*r* = 0.5). If they are of low quality and quality strongly affects group productivity (Fig. B1b), or if they are of high quality and quality weakly affects group productivity (Fig. B1c), then the concession, and hence subordinate fitness, is only slightly reduced by information. By contrast, if subordinates are of low quality and quality weakly affects group productivity (Fig. B19a), or if they are of high quality and quality strongly affects group productivity (Fig. B1d), then the concession is greatly reduced by information and so subordinate fitness would be greatly reduced.

**Fig. 6:**
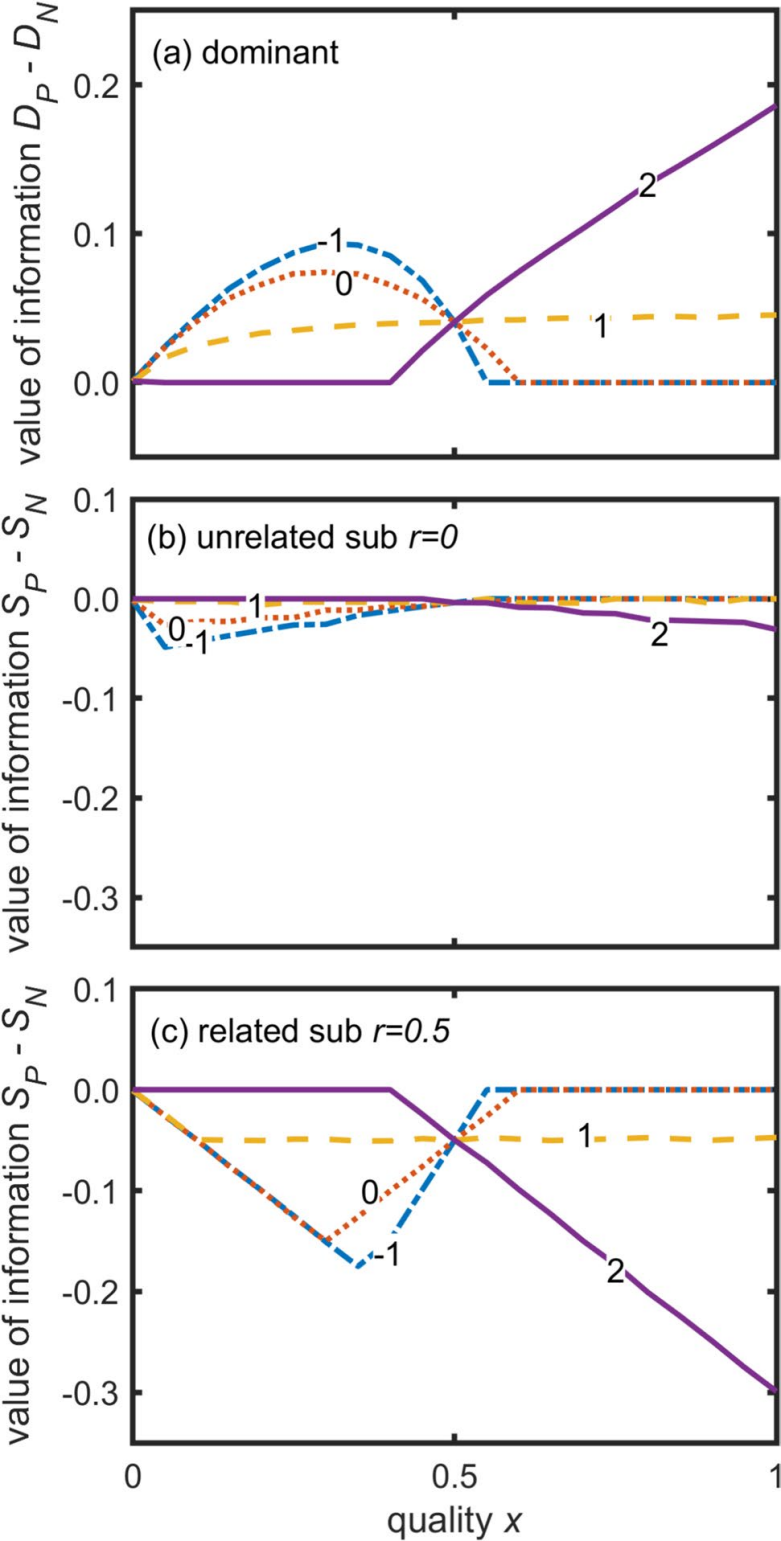
Value of information about relatedness as a function of subordinate quality (horizontal axes) for four values of *a* (lines). The vertical axis is the difference in realised inclusive fitness between the perfect information (*r* known) and the no information case (*r* could be any value). Panels show values of information (**a**) to the dominant and to the unrelated (**b**) or related (**c**) subordinate

The predictions about the value of information about relatedness reflects these differences (Fig. 6). Here, we compare the actual fitness of the individuals for all perceived relatedness values (Fig. 6). The dominant would greatly benefit from information about relatedness if the subordinate is of low quality (*x*) and the relationship between subordinate quality and group productivity (*a*) is weak or negative (dotted and dot-dashed lines), or if *x* is large and *a* is strongly positive (solid and dashed lines) (Fig. 6a). Whilst unrelated subordinates are almost unaffected by the dominant’s information (Fig. 6b), related subordinates show an opposite pattern to the dominant’s but of greater magnitude (Fig. 6a, c): related subordinates get greater fitness if the dominant does not know how related they are.

## Discussion

Using a transactional concession model of reproductive skew with explicit variation and uncertainty, we have shown that the direction of the associations between skew, subordinate outside options and relatedness is influenced by how subordinate quality (outside options) affects group productivity (quality-productivity coefficient; QPC). We have extended the theoretical framework for reproductive skew with two new realistic additions: (i) an association between the subordinate’s outside options and group productivity; and (ii) dominant’s uncertainty about their subordinates. Varying the level of uncertainty (i.e., quantity of information) of dominants about the subordinate’s outside options influenced the optimal concession and fitness outcomes.

Our model predicts that when subordinate quality correlates positively and strongly with group productivity, skew should decrease with subordinate quality; otherwise, skew should increase with subordinate quality. The average concession offered to subordinates always decreases with relatedness, but is greatest when dominants have imperfect information about the subordinate’s relatedness and outside options.

### The importance of the subordinates’ effect on productivity

Empirical tests of the models of reproductive skew have so far yielded inconclusive evidence, as subordinate share has been found variously to increase, decrease or not significantly vary with subordinate outside options, relatedness and group productivity (Nonacs and Hager 2011). Our model revealed that the subordinate’s QPC can alter the sign of the relationships amongst the optimal concession, subordinate quality and relatedness. Dominants maximise their inclusive fitness by retaining low-quality subordinates when the QPC is negative (as those subordinates will provide more help), and high-quality subordinates when the QPC is strongly positive.

Our model predicts that information about kinship influences fitness only when the subordinate’s quality and how this quality translates into group productivity are both high or both low, which might explain the diversity of empirical test results. High-quality subordinates with a low QPC would not get a striking increase in their indirect fitness from helping. Conversely, subordinates who can greatly improve group productivity but have limited outside options may prefer to stay for a low reproductive share, regardless of their relatedness.

Our model shows that simply changing the link between subordinate’s quality and group productivity can switch on (and off) cooperative breeding. When the QPC is high, the optimal share decreases with the (perceived) subordinate quality, which supports the original concession model of skew (Vehrencamp 1983). This finding also matches the prediction of a recent tug-of-war model incorporating variation in the ratio of available breeding sites (comparable to the outside options in our model), in which subordinate share decreased with nest competition when subordinates had the choice between breeding sites (Nonacs 2019). Conversely, when the QPC is low (or negative), the optimal share increases with subordinate quality, in line with the predictions of the restraint model (Reeve and Ratnieks 1993) and suggesting that the QPC might actually determine which individual controls the allocation of reproduction within a transactional framework (Buston et al. 2007). We expect the QPC to be high in conditions where subordinates have limited outside options and group-living is favourable, such as harsh environments rife with outgroup conflict, because individuals would benefit from staying to help until their quality is sufficiently high to disperse and breed (Kokko and Ekman 2002).

Separating the helping effect into the subordinate’s quality and their QPC allows us to disentangle the effects of individual and environmental factors, which may partly explain the variation among and within species in the quality and relatedness of subordinates. Indeed, either high- or low-quality subordinates may be selected to help (Barclay and Reeve 2012). Selection for helping by low-quality subordinates may give rise to two distinct developmental trajectories, helpers or breeders (Fischer et al. 2017).

### Conflict over information about subordinates’ quality

A conflict over the optimal level of information about subordinate quality is predicted, as dominants maximise their fitness at a higher information level than subordinates. Having some information about the subordinate’s quality increases the concession offered by the dominant, as dominants act as though they overestimate the benefits the subordinate will provide, and behave generously to retain the subordinate. Dominants should increase their concession as subordinate quality increases above a certain threshold. The maximal concession should occur when dominants have low levels of information about subordinate quality (unless the subordinate’s QPC is large and positive), as dominants should concede higher reproductive shares. This conflict over the optimal quantity of information is somewhat analogous to models of chick begging, where offspring can conceal their true hunger state to increase parental feeding, in that information is asymmetrical (e.g., Godfray and Johnstone 2000). However, in the parent–offspring signalling game, the parent will not desert the offspring and the aim is to find the optimal parental effort, whereas in our model of skew, groups can break apart. This possibility to leave the interaction partner resembles game-theoretical models of divorce strategies in birds, where individuals pair randomly without any information about their partner’s quality (McNamara et al. 1999). However, in the divorce game, individuals gain perfect information about their partner’s quality after the first breeding season and before deciding whether to stay or leave, whereas in our model the dominant’s level of information remains constant and individuals decide to form a group before the first (and only) breeding season. As in our model, both individuals in the divorce game can decide whether to leave and find a better option (i.e., divorce). High-quality individuals form stable bonds whereas low-quality individuals divorce frequently (McNamara et al. 1999). When both sexes can divorce, high-quality individuals are choosier and divorce less commonly, because both sexes should be less choosy as their probability to be deserted by their partner increases.

An ability to obtain information about the subordinate’s outside options is likely to be selected for when the expected breeding success of a solitary (dominant) breeder is low. This fits with phylogenetic data from the taxa in which cooperative breeding is largely concentrated in harsh, unpredictable environments (Rubenstein and Lovette 2007; Lin et al. 2019), where dominants’ expected solitary breeding success is low and subordinates may vary in quality (e.g., early-life effects: Taborsky et al. 2012). In such situations, dominants would benefit from information about subordinate quality to inform their own reproductive decisions. On the other hand, food scarcity could increase uncertainty by reducing the precision of the perceived information, potentially causing dominants in harsh environments to face higher uncertainty (McNamara et al. 1999; Padamsey et al. 2022). Dominants may then prefer to retain subordinates rather than breed alone, which could favour the evolution of cooperative breeding. Dominants may seek information about subordinates’ quality by exploring the surroundings of the nest (to assess whether potential mates and breeding sites are available) and observing the subordinate to determine its condition (e.g., its size or sexual maturity; Young et al. 2006).

Counteracting this, subordinates may evolve strategies to increase dominants’ uncertainty about their quality. Downplaying apparent quality (e.g., by reducing their helping effort) should be easier for subordinates than pretending to be stronger, as honest signals tend to evolve, because the evolution of honest signalling will tend to constrain the upper limit on work capacity (e.g., Weaver et al. 2018; Wright et al. 2021). Subordinates may also conceal their mating, leading dominants to underestimate their outside options. Indeed, birds are sensitive to others’ visual perspective and can adjust their mating behaviour to keep information private (Arnold 2000). Evidence of this concealing strategy (i.e., sneaky mating) by helpers exists in mammals, birds and fish (Creel et al. 1993; Hellmann et al. 2019; Chen et al. 2021).

### Conflict over information about relatedness

In line with the original concession model of skew, our model predicts that skew should increase with relatedness, which suggests kinship influences reproductive games even when subordinate information and subordinate quality vary (Vehrencamp 1983). While empirical data mostly do not support models of skew within species, skew does increase with average relatedness across species (Nonacs and Hager 2011), at least in birds (Riehl 2017) and social wasps (Oi et al. 2021). Kinship may shape – and hence predict – skew at the between-species level, but it is less clear whether it does so within species (Widdig et al. 2004; Haydock and Koenig 2003; Dugdale et al. 2008; Kaiser et al. 2018).

Our model predicts that dominants will seek information about relatedness that most subordinates are willing to provide, but only up to a point, and only if relatedness is low. A conflict in the optimal information about relatedness emerges with high relatedness. Dominants are selected to concede as little reproductive share as possible and can give less to related subordinates to match the outside option. As a consequence, related subordinates should be selected to withhold information about relatedness, to ensure dominants give them higher reproductive share. The majority of cooperative breeding occurs in family groups (Hatchwell 2009; Rosenbaum and Gettler 2018), which implies high and stable relatedness levels between the helper(s) and the dominant. This low variation in relatedness might be associated with low variation in dominant fitness, which would not select for kin recognition. If we assume low variation in information on one factor limits variation in dominance fitness, we can draw parallels with studies of other types of information. For instance, the choice between different breeding sites might be based on less information if there are fewer available breeding sites, since a model of cooperative breeding found that the variation in fitness across outside options decreases with breeding-site saturation (Nonacs 2019). This indirect investigation of information suggests that the value of information about potential breeding sites is lower when fewer sites are available. Increasing information sampling might therefore only minimally increase fitness. The value of the information about one factor might decrease with the variance in fitness that this factor provides, suggesting that information about relatedness will not be highly valuable in most cooperative breeders (who live in family groups).

Subordinates’ reproductive share decreases as dominants’ information about their relatedness increases, while dominants’ optimal level of information about relatedness decreases with relatedness. Taken together, these predictions reveal a lack of selection for true kin recognition, as subordinates should conceal relatedness and dominants are not strongly selected to acquire it. Most within-species empirical studies suggest that skew does not significantly correlate with relatedness (Nonacs and Hager 2011), which could perhaps be due to true kin recognition not having evolved in these systems. Kin-biased behaviour (i.e., kin discrimination) based on familiarity or shared characteristics such as location or nest odour to discriminate/recognise kin (Holmes and Sherman 1983; Levréro et al. 2015; Charpentier et al. 2020) is well documented (e.g., Komdeur et al. 2004; Mitchell et al. 2018). However, few studies have demonstrated true kin recognition (i.e., phenotype matching) (American toads *Anaxyrus americanus*, pig-tailed macaques *Macaca nemestrina*, mandrills *Mandrillus sphinx;* Holmes and Sherman 1983; Levréro et al. 2015; Rodrigues De Souza et al. 2017; Charpentier et al. 2020). Empirical work found no evidence for kin recognition in house sparrows *Passer domesticus* (Lattore et al. 2019) and dunnocks *Prunella modularis* (Burke et al. 1989) as male breeders did not distinguish their own offspring from others in their care which seems to contradict Hamilton’s rule but is in line with our predictions. A recent model found that stable inaccurate recognition should evolve when the payoff to the interaction partner that benefits from this dishonest signalling is higher than the payoff to both interaction partners when kin recognition is accurate (Sheehan and Reeve 2020). This prediction is in line with our findings that individuals who benefit from errors might select for high uncertainty. Non-discriminating kin may be adaptive if this “veil of ignorance” promotes the redistribution of help to the young that need it most, promoting equality and higher fitness of all group members (Marshall et al. 2021). Another model predicted that in closely related groups, animals would be selected to help without kin discrimination (Duncan et al. 2019). By disentangling kin recognition from relatedness, our model allows us to detect a possible strategy of kin concealment by related subordinates.

### Future tests of the predictions

Our model predicts that the QPC changes the direction of the link between skew and subordinate quality. To test this prediction, future experiments could compare dominants’ acceptance of related subordinates in periods of outgroup conflict (high subordinate QPC) and periods of good environmental quality but high within-group conflict (low subordinate QPC) in two situations: reproductively mature subordinates with several available mates (high quality) and reproductively immature subordinate with no available mates (low quality). Group size may correlate negatively with the QPC, as individuals with similar outside options (quality) help less in larger groups. Here, we predict that older (i.e., higher-quality) subordinates will more often leave the group than younger (lower-quality) subordinates when foraging requires high skill, in large groups and when breeding sites are available, since these conditions are likely associated with low subordinate QPC. In meerkats *Suricata suricatta*, both prey-catching abilities and outside options increase with age (Thornton and McAuliffe 2006; Thornton 2008), and the oldest subordinate females are more often evicted in large groups—where sneaky mating with unrelated males is likely more frequent—than in small groups (Clutton‐Brock et al. 2010). Higher-quality subordinate meerkats therefore leave the group more often in conditions where the QPC appears to be low, which matches our predictions. Further empirical studies should test the predictions by measuring the QPC, subordinate quality and relatedness.

It is clear that information should influence the decisions of the individuals and should therefore be considered where possible in studies of social life. Future empirical studies could potentially test the effect of uncertainty about subordinates’ outside options with experiments that manipulate the quantity of information. This might be feasible in certain species that will breed cooperatively in the laboratory, for example the cichlid *Neolamprologus pulcher* (Braga Goncalves and Radford 2022). For instance, a subordinate without a breeding position and a resident dominant could be placed in adjacent tanks in an observation phase. The subordinate, but not the dominant, would be able to see potential breeding sites (i.e., the subordinate’s outside options) by using an occluder for the dominant. Different experimental treatments would vary the outside options of the subordinate and the visual access of the dominant to those outside options. The dominant and the subordinate would then be given the opportunity to form a group (or not) and breed, to measure skew and cooperative breeding. The dominant could also be given erroneous information, such as via a screen that displays a different number (or quality) of breeding sites, to test the effect of the quantity of information on skew and group formation.

Future theoretical work should explore the effect of relaxing other assumptions, to continue the effort to incorporate more biological complexity into the models. It would be interesting, for instance, to build a model that is not a one-round sequential game and where individuals can acquire information gradually. Future work could investigate which strategies evolve when additional groups members (e.g. those with no outside options (*x* = 0)) provide no benefit or even decrease group productivity, by setting *m* = 0 or *m* < 0 (instead of *m* = 0.35). For now, our modelling has highlighted the need to disentangle ambiguous empirical findings by incorporating unexplored relationships between different drivers that can influence skew (such as the QPC). Taken together with previous findings (Kokko 2003; Ackay et al. 2012), our modelling suggests that in concession models of skew, cooperative breeding can evolve if only dominants have uncertainty about subordinate quality. Our study demonstrates the influence of incorporating variation and uncertainty on model predictions, which supports the argument to add complexity to models to get better theoretical insights (McNamara 2013).

## Supplementary information

Below is the link to the electronic supplementary material.

ESM 1 (DOCX 611 KB)

ESM 2 (DOCX 552 KB)

## Data Availability

The MATLAB code of the models, used to generate the predictions, is available on Dryad: 10.5061/dryad.66t1g1k4h
