# Supplementary material for: Cooperative breeding and the selection for information sharing among groupmates
Source: Behav Ecol Sociobiol. 2025 Jun 17;79(6):70. doi: 10.1007/s00265-025-03604-5 (PMC12170742; doi:10.1007/s00265-025-03604-5)
Supplement: Supplementary file 1 — (DOCX 611 KB) [file 265_2025_3604_MOESM1_ESM.docx]

**APPENDIX for *Cooperative breeding and the selection for information sharing and use* in Behavioral Ecology and Sociobiology**

Laure A. Olivier^1^, Tim W. Fawcett^1^, Andrew N. Radford^2^, Andrew D. Higginson^1^

1: Centre for Research in Animal Behaviour, College of Life and Environmental Sciences, University of Exeter, Exeter EX4 4QG, UK.

2: School of Biological Sciences, Life Sciences Building, 24 Tyndall Avenue, Bristol BS8 1TQ, UK.

Author for correspondence: laure.a.olivier@gmail.com

**APPENDIX A: ANALYSES OF THE EFFECT OF INFORMATION ABOUT SUBORDINATE QUALITY**

To assess the effect of uncertainty about subordinate quality, we built a numerical and an analytical model. The analytical model finds the optimal concession and the conditions where individuals should form groups, by solving mathematically closed form solutions. This is only possible when dominants have either perfect or no information, so we investigated the effect of dominants’ intermediate levels of information about subordinate’s quality numerically (see Appendix B). We included zero or perfect information in this analysis to validate the numerical model by comparison to the analytical results.

Our model developed the well-studied concession model (Johnstone, 2000; Verhencamp, 1983) in which the dominant must concede sufficient share of the reproduction to induce the subordinate to stay, and has perfect information about the subordinate’s outside option *x*¸ which we assume to be determined by their individual quality (hereafter *quality*). The dominant is assumed to be in complete control of the reproductive share, but the subordinate may choose to leave in response. Subordinates vary in the direct fitness payoff associated with *x* which affects the payoff they require to stay in the group. For an overview of the parameters and variables see Table A1.

Table A1 Description of the variables in the analytical model and their baseline values

| **Symbol** | **Definition** | **Values** |
| --- | --- | --- |
| ***Parameters*** |  |  |
| *b* | Direct fitness of a solitary dominant breeder | 1.0 |
| *a* | Quality-productivity coefficient (QPC): effect of subordinate’s quality on group fecundity | 1.0 |
| *r* | Relatedness between the dominant and the subordinate | 0.5 |
| *m* | Minimal effect of helping on group reproductive output | 0.1 |
| *Ω* | Dominant’s information about subordinate’s quality | 0> *Ω>+∞* |
| *θ* | Beta-weighted frequency distribution of *x* from *Ω* |  |
|  | ***Variables of the dominant*** |  |
| *d_A_* | Dominant’s inclusive fitness when alone |  |
| *d_C_* | Dominant’s inclusive fitness when cooperatively breeding |  |
| *d_f_* | Dominant’s direct fitness when cooperatively breeding |  |
| *d_p_* | Dominant’s inclusive fitness when they have perfect information about *x* |  |
| *y* | Reproductive share offered to subordinate by dominant | 0≥*y*≥1 |
| *y_crit_* | Share above which subordinate will help |  |
|  | ***Variables of the subordinates*** |  |
| *s_A_* | Subordinate’s inclusive fitness when alone |  |
| *s_C_* | Subordinate’s inclusive fitness when cooperatively breeding |  |
| *s_f_* | Subordinate’s direct fitness with a dominant |  |
| *x_i_* | Quality of subordinate *i* (i.e. direct fitness if breeding independently) | 0>*x*>*b* |
| *h_i_* | Effect of subordinate help on group reproductive output | *H = ax_i_ + m* |
| *x*_0_* | Quality of subordinate whose payoffs are equal at *y* = 0 |  |
| *x*_y_* | Quality of subordinate whose payoffs are equal at *y** |  |
| *x_crit_* | Quality of subordinate whose options have equal payoff for the dominant |  |

We are interested in the case where *x* varies among subordinates and the dominant payoff is the expected value over the range of possible subordinates. Dominants have access to either perfect information or no information about subordinates’ quality *x*. We assume that subordinate quality *x* follows a uniform distribution.

The direct fitness required by the subordinate to stay help depends on its quality *x* (Table A1). We assume that the dominant’s quality is *b*. So the payoff (i.e. inclusive fitness) to the dominant and subordinate that are symmetrically related with proportion *r* if they don’t stay together are respectively

$d_{A}=b+rx$, (A1)

$s_{A}=rb+x$, (A2)

Since *b* scales the group productivity and dominant quality, without loss of generality we assume that *b* is unity in all the results. We assume that subordinate are not higher quality than the dominant, so the maximum value of *x* is also unity. Because *x* is uniformly distributed between 0 and 1, the expected dominant fitness for all *x* is

$D_{A}=\int_{x=0}^{1} b+rx_{i}=b+\frac{r}{2}$ (A3)

We denote the direct fitness when cooperatively breeding of the dominant and subordinate by *d_f_* and *s_f_*, respectively. The cooperatively breeding group is assumed to have greater success than the sum of the solitary values even when *x=*0 by a value *m* so that

$d_{f}+s_{f}=b(1+m+ax_{i})$ (A4)

where *a* controls the association between the subordinate’s quality and their contribution to group productivity, which we named the Quality-productivity coefficient (QPC). Hence, with a subordinate *i* with quality *x*, the direct fitness of a dominant who gives reproductive share *y_i_* is

$d_{f}=b(1+m+ax_{i})(1-y_{i})$ (A5)

and the subordinate is

$s_{f}=b(1+m+ax_{i})y_{i}$ (A6)

Therefore, the inclusive fitness values when cooperatively breeding when they are related by *r* for the dominant and subordinate respectively are

$d_{C}=d_{f}+rs_{f}=(1-y_{i})b(1+m+ax_{i})+ry_{i}b(1+m+ax_{i})$ (A7)

$$=[1+(r-1)y_{i}]b(1+m+ax)$$

$s_{C}=s_{f}+rd_{f}=r(1-y_{i})b(1+m+ax_{i})+y_{i}b(1+m+ax_{i})$ (A8)

$$=[r+(1-r)y_{i}]b(1+m+ax)$$

Clearly, for *r*<1 the dominant fitness decreases as *y_i_* increases, so they should give the smallest share possible to the subordinate to get them to stay, *s_C_*>*s_A_*, provided that *d_C_*>*d_A_*.

Whether the subordinate would stay depends on the concession given to all subordinates *y,* if

$s_{C}>s_{A}\Rightarrow[r+(1-r)y_{i}]b(1+m+ax_{i})>x_{i}+br$ (A9)

**Perfect Information**

Solving (A9) for *y* gives the optimal *y* under perfect information about subordinate quality (i.e. it changes with the real quality *x_i_*)

$y*=\frac{x-br(m+ax_{i})}{b(1-r)(m+ax_{i}+1)}$ (A10)

Note that if *b*=1 and *a=*0 then the group productivity does not depend on the subordinate’s quality and (A11) reduces to the original concession model (Reeve and Ratnieks, 1993), though note that they assume group productivity is *k* and we assume *k*=*m+1* here.

$y*(a=0,b=1)=\frac{x_{i}-rm}{(1-r)(m+1)}$ (A11)

The concession cannot be negative, and the value of *x* at which *y** is zero

$x_{_{0}}*=\frac{bmr}{1-bar}$, (A12)

so any subordinates with lower quality get a greater fitness than they would alone.

For some *a* and *m* at very high *x* the necessary concession is so great that the dominant would be better off alone, so should offer nothing. By solving *dC > dA* (A7 and A1) we find that the dominant would have higher fitness if alone if subordinate quality is greater than

$x_{_{A}}*=\frac{bm}{1-ba}$ (A13)

Otherwise, the concession will increase with *x* (A11). This results in either a negative or positive change in dominant fitness as *x* increases (see Fig. 1e-h, 8e-h in main text). Thus, given a choice between potential subordinates, in some cases dominants would prefer weaker subordinates. By substituting (A11) into (A7) the dominant’s fitness between these limits if the subordine stays is

$d_{C}*(x_{i},y*)=\left[\frac{x_{i}-br(m+ax_{i})}{b(1-r)(1+m+ax_{i})}(r-1)+1\right]b(1+m+ax_{i})$ (A14)

which simplifies to

$d_{C}*(x_{i},y*)=b(1+r)(m+ax_{i})+b-x_{i}$ (A15)

By solving $\frac{\delta d_{C}*}{\delta x_{i}}>0$, we find that dominant fitness increases with *x* if

$a>\frac{1}{b(1+r)}$ (A16)

That is, increasing the quality-productivity coefficient (*a*), the dominant quality (*b*) and the relatedness (*r*) all increase the chance that dominants prefer high quality subordinates. For the values of *b* and *r* used in Fig. A1 this value of *a* is 2/3 (cf. solid lines in Fig. A1g,h where slope changes from negative to positive).

For *m*<0 it would be better for the dominant to be alone than paired with a very weak subordinate (*x*=0), but if *a* is sufficiently large they would pair with higher quality subordinates (Fig. A1j). By solving $\frac{\delta y*}{\delta x}<0$we find that the optimal concession decreases with *x* (Fig. A1j) if

$a>\frac{m+1}{br}$ (A17)

Putting together the above, we find the expected inclusive fitness of dominants when subordinate quality is uniformly distributed between 0 and 1 is

$\overline{d_{P}}=\int_{0}^{x_{0}*} d_{C}(x_{i},0)\text{ }dx+\int_{x_{0}*}^{x_{A}*} d_{C}(x_{i},y*(x_{i}))\text{ }dx+\int_{x_{A}*}^{1} d_{A}\text{ }dx$ (A18)

where the first term is when the concession is zero, the second term is where the concession is the critical concession necessary and the third term is where the dominant gets higher fitness when alone.

Equation (A18) simplifies to

$\overline{d_{P}}=r+2b-\frac{a^{3}m^{2}(ab^{3}+rb^{2}-r[ab-1])}{2b(ab-1)}$ (A19)

**Effect of perfect information on group formation for each quality**

We investigate the effect of perfect information about subordinate quality on inclusive fitness and group formation, as a function of subordinate quality. The dominants’ and subordinates’ inclusive fitness as a function of *x* when *y=y** and dominants have perfect information are respectively (A16)

and

$s_{P}=b(1+m+ax)\left[y*+r(1-y*)\right]$ (A20)

i.e.

$s_{P}=b(1+m+ax)\left[\frac{br+x-br(m+ax+1)}{b(1-r)(m+ax+1)}(1-r)+r\right]$ (A21)

which simplifies to

$s_{P}=br+x=s_{A}$ (A22)

due to the optimisation by the dominant *d_P_* ≥ *d_N_* for all *x.* The group is stable under perfect information only if *d_C_ >d_A_* and under no information only if *s_N_ > s_A_* (Fig. A1e-l). If *d_P_ < d_A_* then the dominant does not allow the subordinate to stay in the group. If *s_P_ < s_A_* then the subordinate does not stay in the group (Fig. A1).


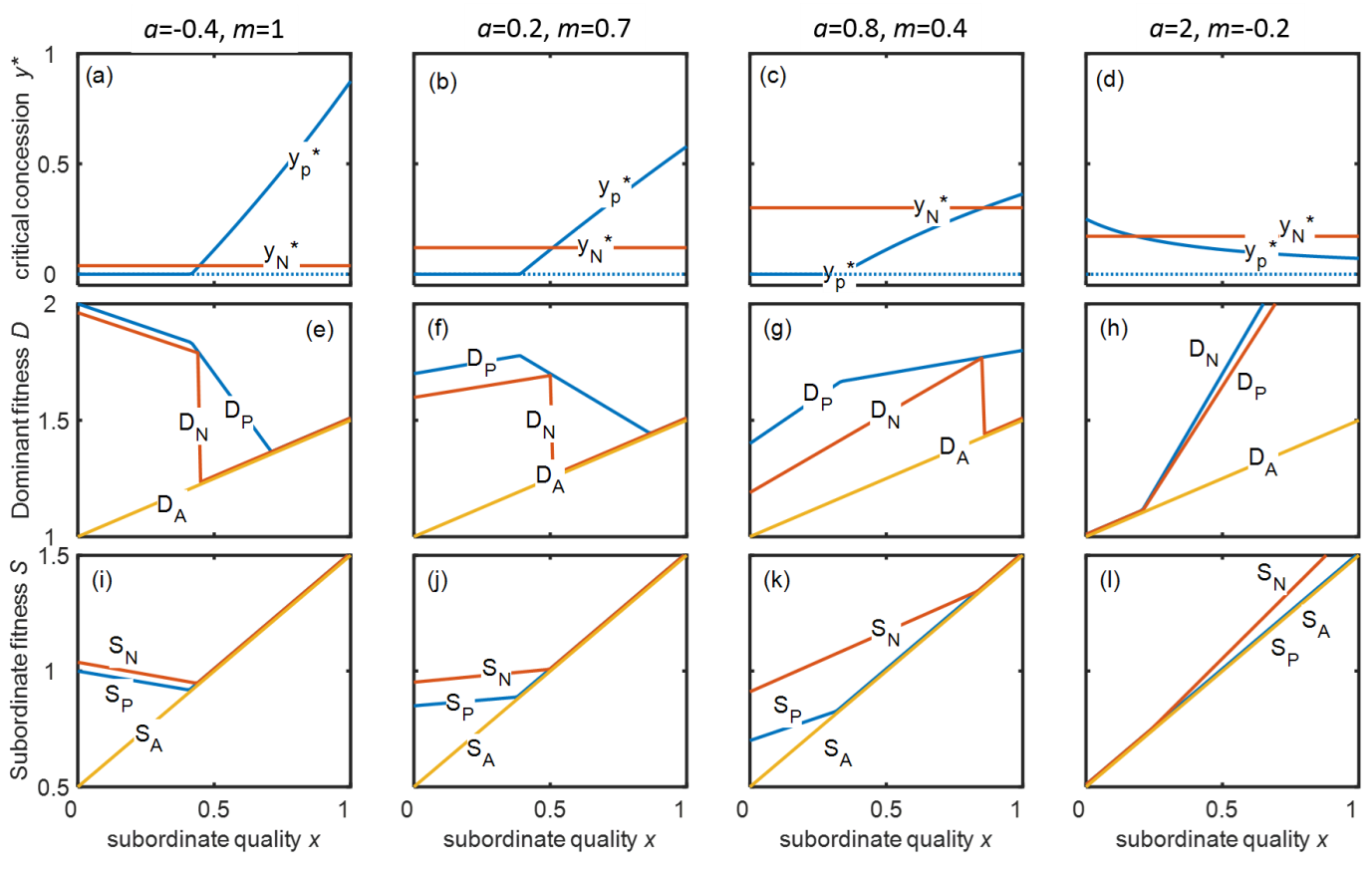


**Fig. A1** Concession (top row), dominant inclusive fitness (middle row), and subordinate inclusive fitness (bottom row) as a function of *x,* for some representative values of *a* and *m* under perfect information P, no information N, and when alone A. The dotted lines indicate *y*=0. The dominant fitness depends on whether the dominant is better with the subordinate than alone (d_P_ >d_A_) and whether the subordinate chooses to stay (s_N_>s_A_). The dominant always does at least as well as no information when they have perfect information, whereas the opposite is true of the subordinate.

The equations become much simpler if the group productivity does not depend on the subordinate quality (*a*=0). The optimal concession simplifies to

$y_{P}*=\frac{x-brm}{b(1-r)(m+1)}$, (A23)

and the *x* at which concession is zero is

$x_{y_{P}*=0}=bmr$ (A24)

The payoff to dominants simplifies to

$d_{C}=b[(1+r)m+1]-x$ (A25)

And the *x* at which the dominant is better off alone is

$x_{_{D_{P}*=D_{A}}}=bm$ (A26)

The expected fitness of the dominant is

$\overline{v_{P}}=\int_{0}^{x_{y_{P}*=0}} d_{P}(x,0)\text{ }dx+\int_{x_{y_{P}*=0}}^{x_{_{D_{P}*=D_{A}}}} d_{P}(x,y_{P}*(x))\text{ }dx+\int_{x_{_{D_{P}*=D_{A}}}}^{1} d_{A}\text{ }dx$ (A27)

$$\overline{v_{P}}=\int_{0}^{bmr} m+1\text{ }dx+\int_{bmr}^{bm} (1+r)m+1-x\text{ }dx+\int_{bm}^{1} 1+rx\text{ }dx$$

$\overline{v_{P}}=b+\frac{r}{2}+\frac{b^{2}m^{2}}{2}\left[1+r-r^{2}\right]$ (A28)

## **No information**

Here, we show how the value of information is affected by individual quality, group productivity and relatedness.We compare the perfect information case to when where dominants have no information about the quality of their potential subordinate. The subordinate quality is only known to be uniformly distributed between 0 and 1. There is a critical quality for any given concession *y*, *x_c_*, below which subordinates would stay and help. That is, the dominant receives help from subordinates with quality 0<*x*<*x*_c_ and breeds alone for those with *x*_c_>*x*>*b*. To solve the optimal reproductive share which maximizes the payoff of the dominant, we integrated it with respect to *x* and then differentiated the integral over the different parts of the reproductive output function of the subordinate (i.e. above and below the threshold of reproductive share), as follows.

The critical value of *x* at which the subordinate would stay depends on the concession given to all subordinates *y* and is the solution for *x* to

$b(1+h)(y+r[1-y])=x+br$ (A29)

which is

$x_{c}(y)=\frac{b(m+1)(y+r[1-y])-br}{1-ab(y+r[1-y])}$ (A30)

We seek the extreme point of the derivative of the fecundity of the dominant with respect to *y* and to *x*, in order to find the maximal and minimal points of the reproductive share function: what is the subordinate quality *x*_min_ to get the minimal reproductive share *y*_min_; likewise what is *x*_max_ where dominant gives maximal *y*_max_.

Thus the general inclusive fitness of uninformed dominants can be found by

$D_{N}=\int_{0}^{x_{c}(y)} D_{P}(x,y)\text{ }dx+\int_{x_{c}(y)}^{1} D_{A}(x)\text{ }dx$ (A31)

which is

$D_{N}=\frac{bx*(2+2m+ax*)\left[ry+1-y\right]}{2}+\frac{2b+r}{2}-\frac{2bx*+r(x*)^{2}}{2}$ (A32)

By solving $\frac{\delta D_{N}}{\delta y}=0$ we find the optimal *y* under no information is $y_{N}*$, but this is too complex to display. We explore this numerically in the main text and in Fig. A2.

**Fig. A2** Fitness and critical quality under no information for representative values of *k* and *m* (*k* shown on lines; *m=*0.8 – *a*/2), as a function of (a) subordinate quality *x* and (b, c) concession *y*. (a) Subordinate fitness if *y=*0.2, (b) Critical quality as a function of concession *y*, (c) Total dominant fitness for all *x* as a function of concession

However, we can make the same simplifications as for the perfect information case of *a*=0. Then

$x*(y)=b(m+1)(y+r[1-y])-br$ (A34)

$D_{N}=\frac{x*b(2+2m)\left[ry+1-y\right]}{2}+\frac{2b+r}{2}-\frac{2bx*+r(x*)^{2}}{2}$ (A35)

$D_{N}=\frac{1}{2}\left[x*(2+2m)\left[ry+1-y\right]+2+r-2x*-r(x*)^{2}\right]$ (A36)

We then differentiate the payoff of the dominant over the reproductive share *y*. We determine the extreme maximum point of this derivative (i.e. maximal fitness; where the derivative is equal to 0) to find the optimal strategy *y**:

$y_{N}*=\frac{m(r^{2}+r-1)}{r-2m+mr+mr^{2}+r^{2}-2}$ (A37)

Thus, after substituting *y** and simplifying, for the optimal reproductive share *y** the optimal fitness of dominant *V** is

$D_{N}*=b+\frac{1}{2}\left[\frac{b^{2}m^{2}}{r+2}+r(b^{2}m^{2}+1)\right]$ (A38)

We can now compare the fitness of the dominant under perfect and no information. The value of knowing quality is

$$D_{P}*-D_{N}*=b+\frac{r}{2}+\frac{b^{2}m^{2}}{2}\left[1+r-r^{2}\right]-b-\frac{1}{2}\left[\frac{b^{2}m^{2}}{r+2}+r(b^{2}m^{2}+1)\right]$$

$$D_{P}*-D_{N}*=\frac{1}{2}\left[r+b^{2}m^{2}\left[1+r-r^{2}\right]-\frac{b^{2}m^{2}}{r+2}-r(b^{2}m^{2}+1)\right]$$

$D_{P}*-D_{N}*=\frac{b^{2}m^{2}}{2}\left[1-r^{2}-\frac{1}{r+2}\right]$ (A39)

which is positive unless *r*>0.8. The value of information always increases as *b* and *m* increase, and by differentiating with respect to *r* we find that the value decreases as *r* increases if *r* > 0.112.

The subordinates’ payoff under perfect information is

$s_{P}=b(1+m)(y*+r[1-y*])$ (A40)

$s_{P}=b(1+m)\left(\frac{x-brm}{b(1-r)(m+1)}(1-r)+r\right)$ (A41)

$s_{P}=x+br$; (A42)

i.e. the solitary payoff since the dominant matches it and if $bmr<x<bm$. Since the higher quality dominants also get this (solitary breeding) we do not need to calculate it.

For low quality individuals for which *y**=0, *x<bmr*, the payoff is

$s_{P}=b(1+m)(0+r[1-0])$ (A43)

$$=b(1+m)r$$

The subordinate payoff under no information if they stay does not depend on *x*

$$s_{N}=b(1+m)\left(\frac{m(r^{2}+r-1)}{r-2m+mr+mr^{2}+r^{2}-2}(1-r)+r\right)$$

$s_{N}=b(m+r)-\frac{bm}{r+2}$ (A44)

To high *x* the benefit of the dominant having perfect information is

$s_{P}*-s_{N}*=x+br-b(m+r)+\frac{bm}{r+2}$ (A45)

$$w_{P}*-w_{N}*=x-bm\left(1-\frac{1}{r+2}\right)$$

Which is positive if

$x>bm\left(1-\frac{1}{r+2}\right)$ (A46)

The critical value of *x* below which the subordinate stays is

$$x*(y*)=b(m+1)(y*+r[1-y*])-br$$

$x*(y*)=\frac{bm(r+1)}{r+2}$ (A47)

The benefit of information occurs for a region where the subordinate would stay if

$bm\left(1-\frac{1}{r+2}\right)<\frac{bm(r+1)}{r+2}$ (A48)

$$bm-\frac{bm}{r+2}<\frac{bm(r+1)}{r+2}$$

$$1-\frac{1}{r+2}<\frac{r+1}{r+2}$$

$$r+2-1<r+1$$

$$1<1$$

i.e. never.

Low quality individuals (*x<bmr*) benefit from no information if

$s_{P}*<s_{N}*=b(1+m)r-b(m+r)+\frac{bm}{r+2}$ (A49)

$s_{P}*<s_{N}*=r(r+1)<1$,

Which is when *r<*0.618.

In summary, high quality individuals do not benefit from the dominant having information about their quality, whereas low quality individuals benefit from the dominant not knowing their quality.

**Imperfect information**

The aim is to calculate dominant and subordinate inclusive fitness and optimal reproductive share as a function of information and subordinate quality *x.* The dominant has a prior belief about the probability distribution of the outside options of a potential subordinate *x*. It chooses a sampling strategy *Ω* to acquire more information about *x*. With this information, it updates its subjective knowledge about *x*: its prior belief becomes a posterior belief. This way of updating information is Bayesian learning (Trimmer et al., 2011). The model systematically tests how the quantity of information about *x* influences the inclusive fitness and the optimal reproductive decision of the dominant by simulating 12 sampling strategies (0 ≤ Ω ≤+∞). We systematically explore group formation for different values of relatedness *r*, QPC *a* and synergy effect *m.*

Bayesian learning occurs when the dominant has imperfect information: the dominant only infers the quality of the subordinate it meets by observing a number *Ω* of times (McNamara, Green, & Olsson, 2006). Dominants who perform the maximal sampling Ω are assumed to have perfect information (i.e. know *x* without error). In this sequential model, the dominant uses a sampling strategy *Ω* and forms a belief about the probability distribution of *x* before deciding which proportion of the total group reproduction *y* to offer to a potential helper. This probability distribution (A52) is weighted by what the dominant knows with the parameters α and β.

$\begin{matrix} \alpha=1+x_{val}+\Omega\\ \beta=1+(1-x_{val})\Omega\end{matrix}$ (A50)

These alpha and beta parameters control the shape of the probability beta-weighted distribution function of the belief of the dominant about the quality of the subordinate. Subordinate’s quality *x* is a probability of successful helping per attempt, where the subordinate can either help or leave. Since each subordinate-dominant interaction only has one outcome, all subordinates’ outside options are independent of each other and each interaction has the same probability of success, *x,* which is a binomial distribution.

We calculate the beta-weighted distribution *θ(x)* of the probability that the dominant perceives each subordinate quality *x* given the real quality *x*

$\theta(x)=x^{\alpha-1}(1-x)^{\beta-1}=P(perceived|actual)$ (A51)

The probability of the perceived subordinate quality, as a function of the quantity of information sampling and the parameters α and β, is

$P(\Omega|\alpha,\beta)=\frac{1}{\theta(\alpha,\beta)}\Omega^{\alpha-1}(1-\Omega)^{\beta-1}I_{[0,1]}(p)$ (A52)

To normalise the weighted beta distribution, we divide each beta-weighted distribution by the sum of the beta-weighted distribution so it sums to unity. As sampling *Ω* gets very large the distribution gets increasingly narrow; i.e. perfect information.

Given the probability of each perceived *x*, we can calculate the probability of each actual *x* given the perception

$P(actual|perceived)=P(perceived|actual)\frac{P(actual)}{P(perceived)}$ (A53)

We store the perceived and actual inclusive fitness, since dominants with limited information cannot know exactly what payoff they will get.

We create a weighted payoff to the dominant for each share *y*, because the probability to meet a subordinate of a given quality is a function of the position of the quality on the distribution function. Dominants base their decision on all subordinates weighted by the probability on the probability of each actual given their perceived distribution *θ* (perceived fitness), although their actual payoff is from this particular subordinate. We calculate dominants’ perceived inclusive fitness for each *x* and *y*, as a function of the expected decision of the subordinate, as was done in the No Information case.

We calculate actual inclusive fitness by determining if subordinates of the possible qualities stay with *y*_Imperfect_* or leave weighted by their probabilities. Note that if the payoff from solitary breeding is higher than that of breeding cooperatively, then the dominant gets the same payoff if it has perfect information as if it makes errors, which suggests that information about a potential subordinate is not valuable when the dominant prefers solitary breeding.

When group productivity strongly depends on subordinate quality and weak subordinates reduce productivity (Fig. A3), dominants avoid accepting low-quality subordinates. They therefore have a threshold of perceived quality below which they would evict the subordinate. For less than perfect information (Fig. A3a,b,c) the threshold of maximal quality for which subordinate will stay, and the threshold of quality below which dominant will evict them, divide the (*x,z*) space into quadrants where both, neither or one of the individuals want to form a group. With more information *y** decreases as *z* increases (above a minimum) which results in an increasing subordinate’s threshold: when *z<x* subordinates do not join the group as the concession is insufficient.

When subordinate quality negatively affects group productivity – perhaps by increasing conflict or if strong subordinates require more food but don’t work harder – then dominants should evict strong subordinates. For no or some information the subordinate’s and dominant’s thresholds divide the (*x,z*) space into quadrants where both, neither or one of the individuals want to form a group (Fig. A3). For high information there is a band in the *z* dimension where the dominant does not want the subordinate to join, but has a high concession so that lower-quality subordinates will try to join.

**
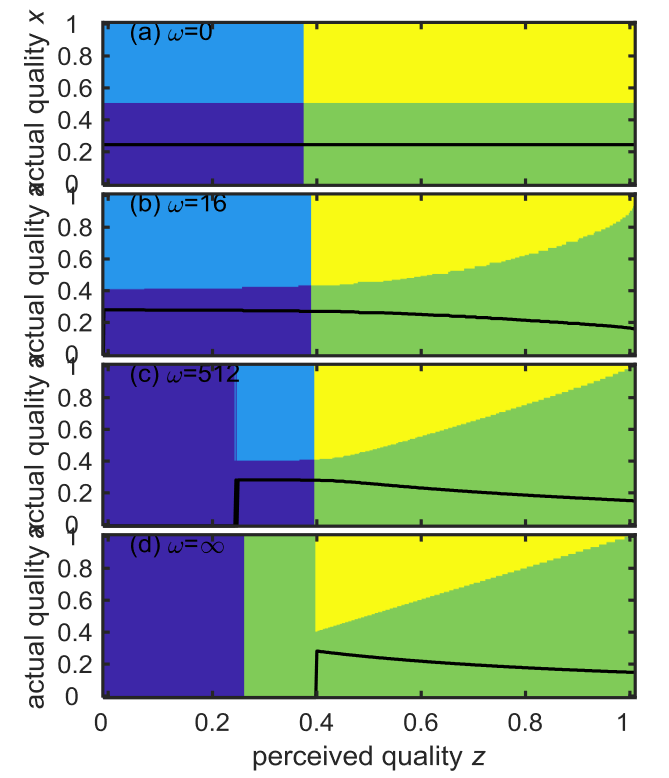
**

**Fig. A3** As Fig. 2 (see main text) but where group productivity strongly increases with subordinate quality and subordinates are more highly related (*a*=2, *m*=-0.4, *r*=0.5). The colours show the areas of perceived quality by dominant (horizontal axis) and actual quality of subordinate (vertical axis) where for the optimal *y* (green) both dominant and subordinate would do better in a group *; (yellow) only the dominant would do better in a group; (cyan) only the subordinate would do better in a group; (dark blue) neither would do better in a group. Parameter values: *a*=0.5, *m*=0.35, *r*=0.25. Note that *m*<0 so that dominants avoid accepting poor quality subordinates. They therefore have a threshold of minimal subordinate quality along the *z* axis

**
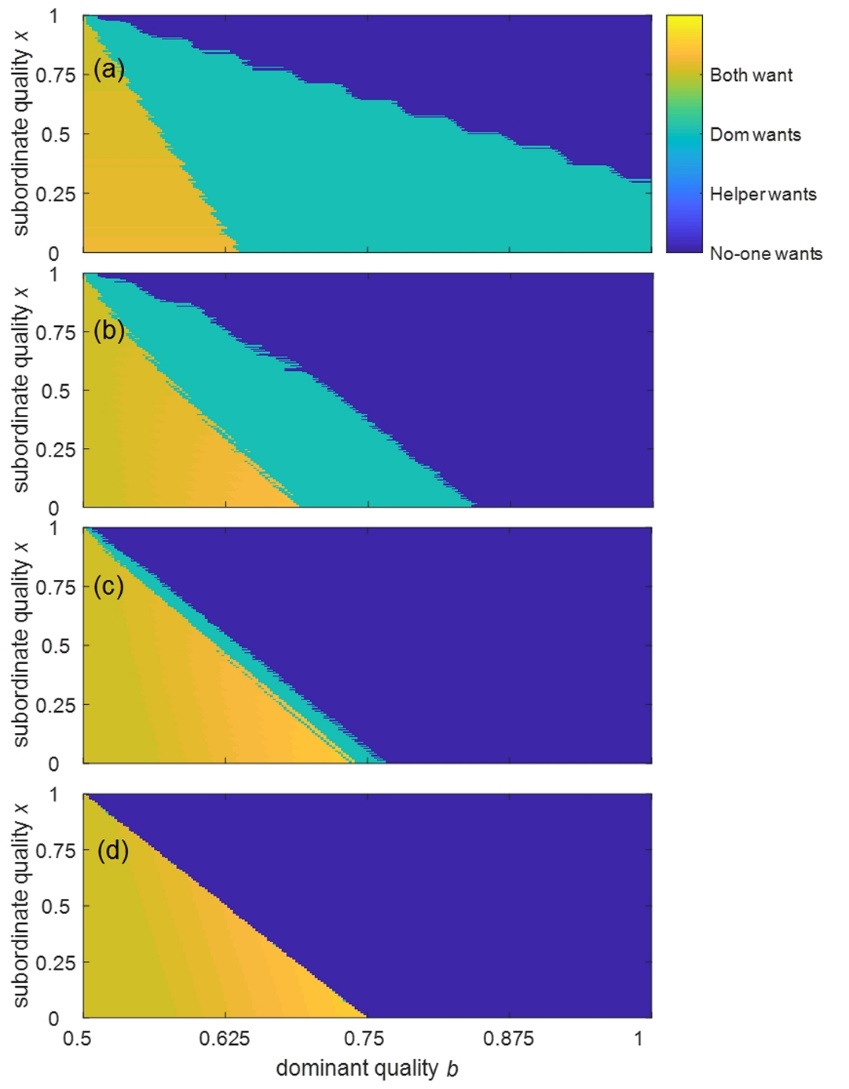
**

**Fig. A4** Group formation as function of outside option *x* and benefit of cooperation *m*. Comparison of the numerical model where dominants have no information to a previous model that assumed symmetrical lack of information (Akçay et al. 2012). In order to compare directly we varied dominant quality b (0.5≤*b*≤1) and subordinate quality *x* (0 ≤ *x* ≤ 1), set *a* = 0 and fixed the group productivity $b\left( 1+m+ax \right)=1$ and by and fixing $m=\frac{1}{b}-1$. Optimal decisions regarding cooperative breeding as a function of subordinate and dominant qualities, for increasing quality-productivity coefficient.
