# Supplementary material for: Cooperative breeding and the selection for information sharing among groupmates
Source: Behav Ecol Sociobiol. 2025 Jun 17;79(6):70. doi: 10.1007/s00265-025-03604-5 (PMC12170742; doi:10.1007/s00265-025-03604-5)
Supplement: Supplementary file 2 — (DOCX 552 KB) [file 265_2025_3604_MOESM2_ESM.docx]

**APPENDIX for *Cooperative breeding and the selection for information sharing and use* in Behavioral Ecology and Sociobiology**

Laure A. Olivier^1^, Tim W. Fawcett^1^, Andrew N. Radford^2^, Andrew D. Higginson^1^

1: Centre for Research in Animal Behaviour, College of Life and Environmental Sciences, University of Exeter, Exeter EX4 4QG, UK.

2: School of Biological Sciences, Life Sciences Building, 24 Tyndall Avenue, Bristol BS8 1TQ, UK.

Author for correspondence: laure.a.olivier@gmail.com

**APPENDIX B: NUMERICAL MODEL INVESTIGATING UNKNOWN RELATEDNESS**

We systematically explored how dominants’ uncertainty about their relatedness to the subordinate influences the optimal concession. This numerical model is based on the concession model of skew.

**Table B1**: Description of the variables specific to the numerical model of unknown relatedness and their baseline values. For other variables see Table A1 in Appendix A

| Symbol | Definition | Values |
| --- | --- | --- |
| *r* | Relatedness between the dominant and the subordinate | [0-1] |
| *r** | Critical value of relatedness |  |
| ** | Relatedness above which the concession is zero |  |
| *r*(y)* | Critical relatedness below which subordinates would not stay, for a given concession *y* |  |
| *y*_N_* | Optimal concession to all possible subordinates when dominant has no information about relatedness |  |
| *y’* | Change in concession given information about *r* |  |

**Asocial**

The expected fitness when alone for the dominant is

$\overline{v_{A}}=\int_{0}^{r*} b+rx\text{ }dr$ (B1)

$$\overline{v_{A}}=\left[br+r^{2}x\text{ }\right]_{0}^{r*}$$

$$\overline{v_{A}}=br*+\frac{r*^{2}x}{2}\text{ }$$

**Perfect information**

What is the effect of information when relatedness is uncertain? Since *x* is a constant, we don’t need to show *x, a,* or *m*.

$s_{C}>s_{A}\Leftrightarrow b(1+h)(y+r[1-y])>br+x$, (B2)

The optimal concession is

$y*=\frac{x-bhr}{b(1-r)(1+h)}$ (B3)

The optimal concession declines as *r* increases, so the relatedness above which the concession is zero is

$r_{y_{P}*=0}<\frac{x}{bm}$, (B4)

The fitness of the dominant with a subordinate is

$d_{C}=b[(1+r)m+1]-x$ (B5)

When cooperating better than alone for the dominant

$d_{C}>d_{A}\Leftrightarrow b[(1+r)m+1]-x>b+xr$ (B6)

$$r*>-1$$

which is always the case. The change in the difference between cooperative and asocial fitness for the subordinate with respect to *r* is

$\frac{d}{dr}=b(1+m)[1-y]-b$ (B7)

which is positive provided

$y<\frac{m}{m+1}$ (B8)

Since *y*<0.5, the dominant won’t concede more than half the reproduction; this will always occur if *m*>1. Therefore, under most situations the more related the subordinate is, the more they are inclined to stay. This is why *y** decreases as *r* increases.

Dominant fitness over a range of subordinate relatedness is

$\overline{v_{P}}=\int_{0}^{r_{y_{P}*=0}} d_{P}(r,y_{P}*(r))\text{ }dr+\int_{r_{y_{P}*=0}}^{r*} d_{P}(r,0)\text{ }dr$ (B9)

$\overline{v_{P}}=br*(m+1)-\frac{x^{2}}{2bm}$ (B10)

**No information**

The critical relatedness below which they would not stay is

$r*(y)<1+\frac{x-bm}{b[m-y(m+1)]}$ (B11)

So the total dominant fitness is

$\overline{v_{N}}=\int_{0}^{r*} s_{A}(r)\text{ }dr+\int_{r*}^{R} s_{P}(r,y)\text{ }dr$ (B12)

$\overline{w_{N}}=\left[\frac{r^{2}x+2br}{2}\right]_{0}^{r*(y)}+\left[\frac{b(m+1)[r^{2}y+2r(1-y)]}{2}\right]_{r*(y)}^{R}$ (B13)

The optimal concession to all possible subordinates is

$y_{N}*=\frac{1}{3}-\frac{b-2x}{3b(1+h)}$ (B14)

The change in concession given information is

$y'=y_{N}*-y_{P}*=\frac{x-bhr}{b(1-r)(1+h)}-\frac{1}{3}+\frac{b-2x}{3b(1+h)}$ (B15)

$$y'=\frac{(bh-x)(2r+1)}{3b(1-r)(1+h)}$$

The value of knowing relatedness for the dominant is

$d_{P}*-d_{N}*=bR(m+1)-\frac{x^{2}}{2bm}-br*(m+1)+\frac{x}{2}-\frac{bm(1+r*^{2})}{2}+xr*$ (B16)

$$d_{P}*-d_{N}*=x\left(r*+\frac{1}{2}\right)-\frac{x^{2}}{2bm}-\frac{bm(1+r*^{2})}{2}$$

The change in subordinate fitness when dominant knows relatedness (see Fig. 6 in main text) is

$\begin{matrix} b(1+h)[y'+r(1-y')]+br+x \\ =(b+bh)[\frac{(bh-x)(2r+1)}{3b(1-r)(1+h)}+r(1-\frac{(bh-x)(2r+1)}{3b(1-r)(1+h)})] \\ =\frac{bm}{3}-\frac{x}{3}+br-\frac{2rx}{3}+\frac{abx}{3}+\frac{5bmr}{3}+\frac{5abrx}{3} \\ =\frac{1}{3}(bm-x+3br-2rx+abx+5bmr+5abrx) \end{matrix}$ (B17)

The value of information to the subordinate is never positive contrary to the value of information to the dominants, consequently subordinates should not signal their relatedness (Fig. 6b, c in main text). Hiding strategies might evolve. The information is most harmful to a related subordinate with very high quality and QPC=2 (Fig. 6c in main text).

The value of information to the dominant is maximal for low-medium subordinate quality when the quality-productivity coefficient is low, but increases with subordinate quality when QPC is high (Fig. 6a in main text).

Dominant information about relatedness does not change subordinate fitness when *x* is

$x=\frac{bm+3br+5bmr}{2r-ab-5abr+1}$ (B18)

When *b*=1, which is the baseline value in our models, this simplifies to

$x=\frac{m+3r+5mr}{2r-a(5r+1)+1}$ (B19)

Or when *a* is

$a=\frac{-bm+x-3br+2rx-5bmr}{bx+5brx}$ (B20)

When *b*=1 and *r*=0.5 as in diploid families, a simplifies to

$a=\frac{-7m-4x+3}{7x}$ (B21)

When *m*=0.1, dominant’s level of information about relatedness does not change subordinate fitness if *a*~=0.0857. So subordinate quality’s influence on helping effect is negligible when subordinate fitness is not influenced by information about relatedness. The change in subordinate fitness when dominant knows relatedness increases as *a* increases. The value for subordinate of signalling or not their relatedness depends on how their help translates into helping, *a*.


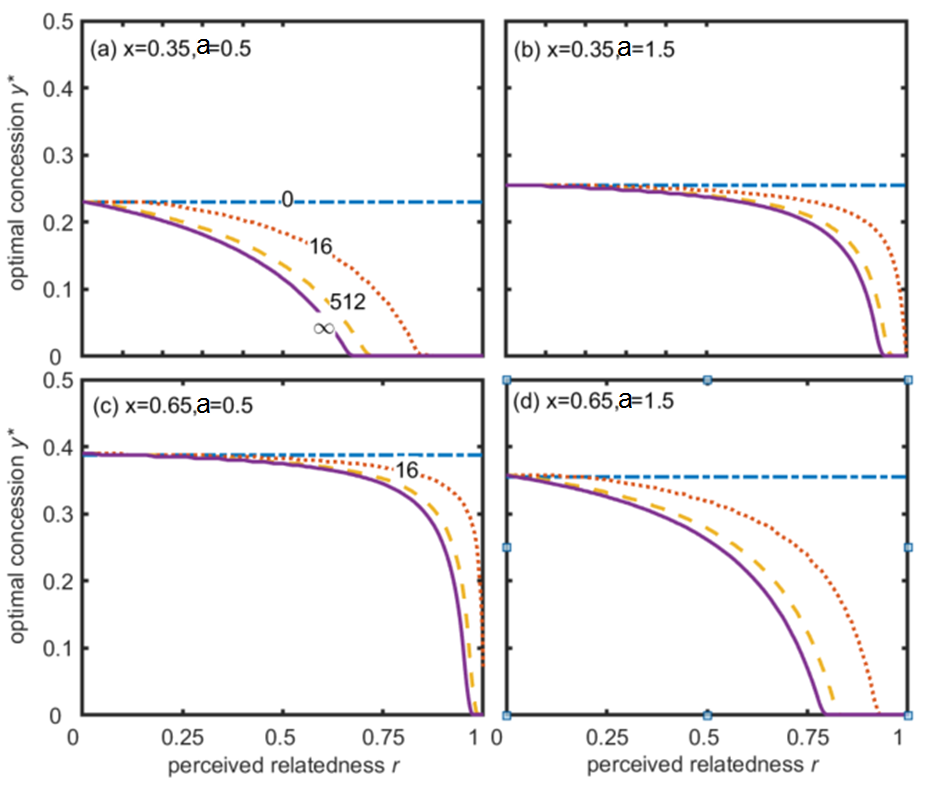


**Fig. B1** Effect of perceived relatedness on the optimal concession *y** for 4 values of information (lines): no information about relatedness *Ω* =0 (dot-dash line), some information *Ω* =16 (dotted line), high information *Ω* =512 (dashed line), perfect information *Ω* =∞ (solid line). Panels show (a, b) low and (c, d) high quality subordinate and (a, c) weak and (a,b, d) strong dependence of group productivity on subordinate quality. In all cases and for all relatedness values, the concession declines as relatedness and information increase


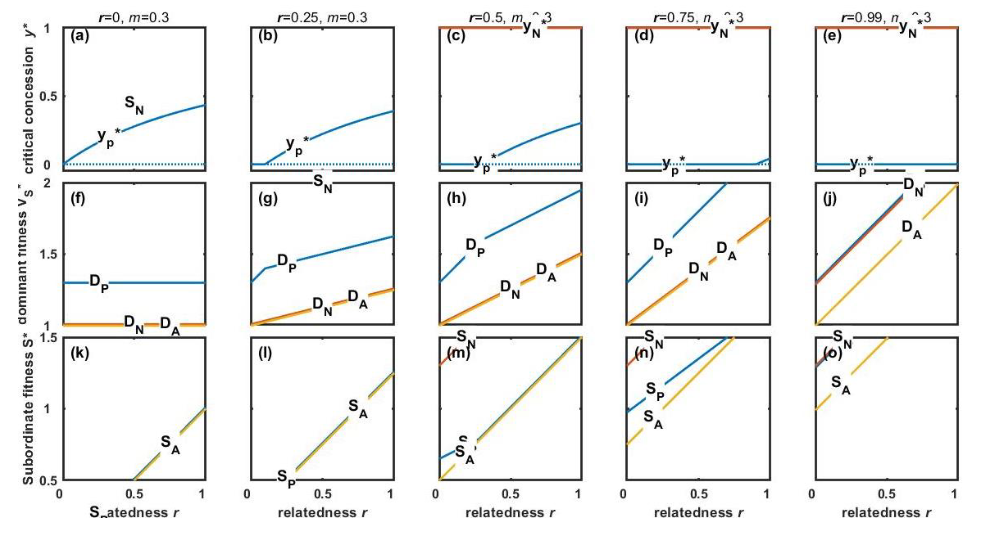


**Fig. B2** Optimal concession, subordinate fitness and dominant fitness as a function of relatedness,

when dominant have perfect information (P), no information (N) or imperfect information (I) about

subordinate relatedness, and when they breed alone (A). Parameter values: *b*=1, *a*=1, *m*=0.3, *r*=0.5
